# Emerging Biosensing Methods to Monitor Lung Cancer Biomarkers in Biological Samples: A Comprehensive Review

**DOI:** 10.3390/cancers15133414

**Published:** 2023-06-29

**Authors:** Raja Chinnappan, Tanveer Ahmad Mir, Sulaiman Alsalameh, Tariq Makhzoum, Alaa Alzhrani, Khalid Alnajjar, Salma Adeeb, Noor Al Eman, Zara Ahmed, Ismail Shakir, Khaled Al-Kattan, Ahmed Yaqinuddin

**Affiliations:** 1 College of Medicine, Alfaisal University, Riyadh 11533, Saudi Arabia; salsalameh@alfaisal.edu (S.A.); aimalzahrani@kau.edu.sa (A.A.); snadeeb@alfaisal.edu (S.A.); naleman@alfaisal.edu (N.A.E.); zaahmed@alfaisal.edu (Z.A.); ishakir@alfaisal.edu (I.S.); kkattan@alfaisal.edu (K.A.-K.); ayaqinuddin@alfaisal.edu (A.Y.); 2 Laboratory of Tissue/Organ Bioengineering & BioMEMS, Organ Transplant Centre of Excellence, Transplant Research & Innovation Department, King Faisal Specialist Hospital and Research Centre, Riyadh 11211, Saudi Arabia; 3 Medical Laboratory Technology Department, Faculty of Applied Medical Sciences, King Abdulaziz University, Jeddah 21589, Saudi Arabia

**Keywords:** lung cancer, biosensors, lung cancer biomarkers, DNA methylation, microRNA, point of care testing, self-health monitoring, aptasensors

## Abstract

**Simple Summary:**

Lung cancer causes more than 1.5 million deaths every year around the globe. Among all cancer types, lung cancer is one of the most common. More than 75% of the cases are identified only in the advanced stage due to poor prediction in the early stage. There are many traditional methods used for the diagnosis of lung cancer, however, they are not very accurate to predict lung cancer in the early stage and are highly expensive. Therefore, developing a more accurate, low-cost, and rapid method is crucial for early-stage detection of lung cancer. Biosensors are developed using advanced modern technologies and nanomaterials. They are robust, low-cost, more accurate, and less time-consuming for sample analysis. Moreover, it can be handled by untrained persons. In this review article, we discussed various analytical methods for the development of biosensors for the sensitive diagnosis of different kinds of lung cancer biomarkers. The major challenges and prospects for the application of biosensors in point-of-care testing have been discussed.

**Abstract:**

Lung cancer is the most commonly diagnosed of all cancers and one of the leading causes of cancer deaths among men and women worldwide, causing 1.5 million deaths every year. Despite developments in cancer treatment technologies and new pharmaceutical products, high mortality and morbidity remain major challenges for researchers. More than 75% of lung cancer patients are diagnosed in advanced stages, leading to poor prognosis. Lung cancer is a multistep process associated with genetic and epigenetic abnormalities. Rapid, accurate, precise, and reliable detection of lung cancer biomarkers in biological fluids is essential for risk assessment for a given individual and mortality reduction. Traditional diagnostic tools are not sensitive enough to detect and diagnose lung cancer in the early stages. Therefore, the development of novel bioanalytical methods for early-stage screening and diagnosis is extremely important. Recently, biosensors have gained tremendous attention as an alternative to conventional methods because of their robustness, high sensitivity, inexpensiveness, and easy handling and deployment in point-of-care testing. This review provides an overview of the conventional methods currently used for lung cancer screening, classification, diagnosis, and prognosis, providing updates on research and developments in biosensor technology for the detection of lung cancer biomarkers in biological samples. Finally, it comments on recent advances and potential future challenges in the field of biosensors in the context of lung cancer diagnosis and point-of-care applications.

## 1. Introduction

Globally, lung cancer is one of the leading causes of cancer-related deaths, killing more than 1.8 million people annually [1]. Although the primary cause of lung cancer is tobacco consumption, the prevalence of lung cancer among nonsmokers is also increasing, and genetic and environmental factors are believed to play a significant role in susceptibility to lung cancer. Smoking cessation is strongly recommended in the United States for all active smokers, especially those undergoing lung cancer screening and treatment. As a result, both lung cancer incidence and mortality rates have declined, which may be attributed to successful counseling efforts to reduce smoking. Globally, however, both tobacco consumption and lung-cancer-linked abnormalities are increasing, a trend that is skewed toward underdeveloped countries. Currently, over 50% of new cancer cases occur in developing countries [2]. Of particular concern are risk factors related to environmental contaminants such as radon exposure, air pollution, secondary smoking, and asbestos [2,3]. The impact of non-tobacco factors is best demonstrated in Chinese women, where the prevalence of lung cancer is similar to many European countries, despite their lower smoking incidence [2]. However, as the largest risk factor, the majority of efforts in the prevention of lung cancer continue to consist of the prevention of adolescents from picking up smoking and increasing smoking cessation [2,3,4]. Lung cancer is classically grouped into two subgroups based on histological findings: non-small-cell lung cancer (NSCLC) and small-cell lung cancer (SCLC). Small-cell lung cancer is characterized by small cells with scanty cytoplasm and numerous mitotic figures (a process of dividing to create two new cells) [5]. It is associated with a very bad prognosis among lung cancers due to high rates of metastasis; therefore, it is typically managed with chemotherapeutic agents only [4]. Surgical treatment is potentially considered for stages 1 and 2, though the evidence for surgery in randomized controlled clinical trials (RCTs) is mixed. However, adjuvant chemotherapy and radiotherapy have been shown to potentially improve survival chances. In advanced stages of SCLC, treatment is primarily palliative because of the dismal prognosis and overall survival. Therapy usually results in an overall survival benefit of less than one year. Additionally, relapse tends to be common [4].

NSCLC is typically subcategorized into adenocarcinoma, squamous cell carcinoma, and large cell carcinoma based on tumor morphology and immunohistochemical staining.

Following diagnosis and TNM staging, treatment can be initiated. When a tumor is potentially resectable, for example, in early-stage carcinomas, the primary focus of treatment is usually surgical resection, while radiotherapy and radiofrequency ablation are usually reserved for high-risk patients [3]. Adjuvant chemotherapy has clear indications in postoperative stages 2 and 3, whereas in stage 1, its usefulness is unclear, and neoadjuvant chemotherapy has not been widely evaluated. For locally advanced tumors that are unresectable, chemoradiotherapy is the optimal treatment and can be followed by chemotherapy followed by radiation therapy. The clinical presentation has long been emphasized as the first step toward diagnosis. Lung cancer patients display respiratory and constitutional symptoms such as cough, dyspnea, and hemoptysis. They may also present with nerve compression, leading to hoarseness or breathlessness, superior vena cava compression, or severe chest pain due to pleural or septal invasion and lymph node involvement [6]. Usually, patients do not visit the hospital immediately after noticing symptoms, which limits their usefulness as diagnostic and screening tools. However, when patients do visit the hospital, whether for routine clinical evaluation or due to increased severity of symptoms, it can be helpful in raising suspicion of a neoplastic process. Similarly, laboratory tests can also suggest malignancy, but they are generally non-specific and are more useful as prognostic indicators rather than diagnostic ones [6,7]. On the other hand, imaging studies, especially CT scans, play a very useful role in diagnosis, not only in suggesting a malignant process but also helping to focus attention on the lungs in patients with unspecific symptoms [5,7]. Additionally, recent advancements in artificial intelligence and computer-aided diagnostics approaches have gained tremendous attention as screening, diagnostic, and follow-up tools [8,9]. Ultimately, confirmation of the diagnosis requires a biopsy, where the morphology of the cancer is observed, along with potential staining techniques to confirm cell type [10,11].

Unfortunately, the prognosis of lung cancer remains relatively abysmal due to diagnoses often not occurring until the late stages of the disease [1,12]. This is in part due to patients’ hesitancy to report symptoms and delayed referral resulting from the low specificity of common symptoms [6]. For this reason, early disease detection and screening methods are also being explored. Low-dose computed tomography (LDCT) was tested in national lung screening trials in 2011, which showed a 20% reduction in mortality when high-risk patients were screened, leading to the implementation of annual LDCT for high-risk adults in the US [13]. Similar RCTs have shown the success of LDCT in Italy and Germany [14,15]. Despite this, European nations have yet to adopt similar recommendations [3,4].

Because lung cancer tends to be a serious and incurable disease, there is an urgent need for rapid and effective diagnostic modalities that are both inexpensive to implement in point-of-care diagnostics and do not require trained specialists. The design of novel substrates and the development of highly sensitive sensors offers hope in the identification of cases in the pre-malignant and pre-metastatic stages. With their high sensitivity, high selectivity and high-throughput detection capabilities, biosensors can rapidly contribute to the early detection, diagnosis, and prevention of disease and mortality [16,17].

**Scheme 1:**
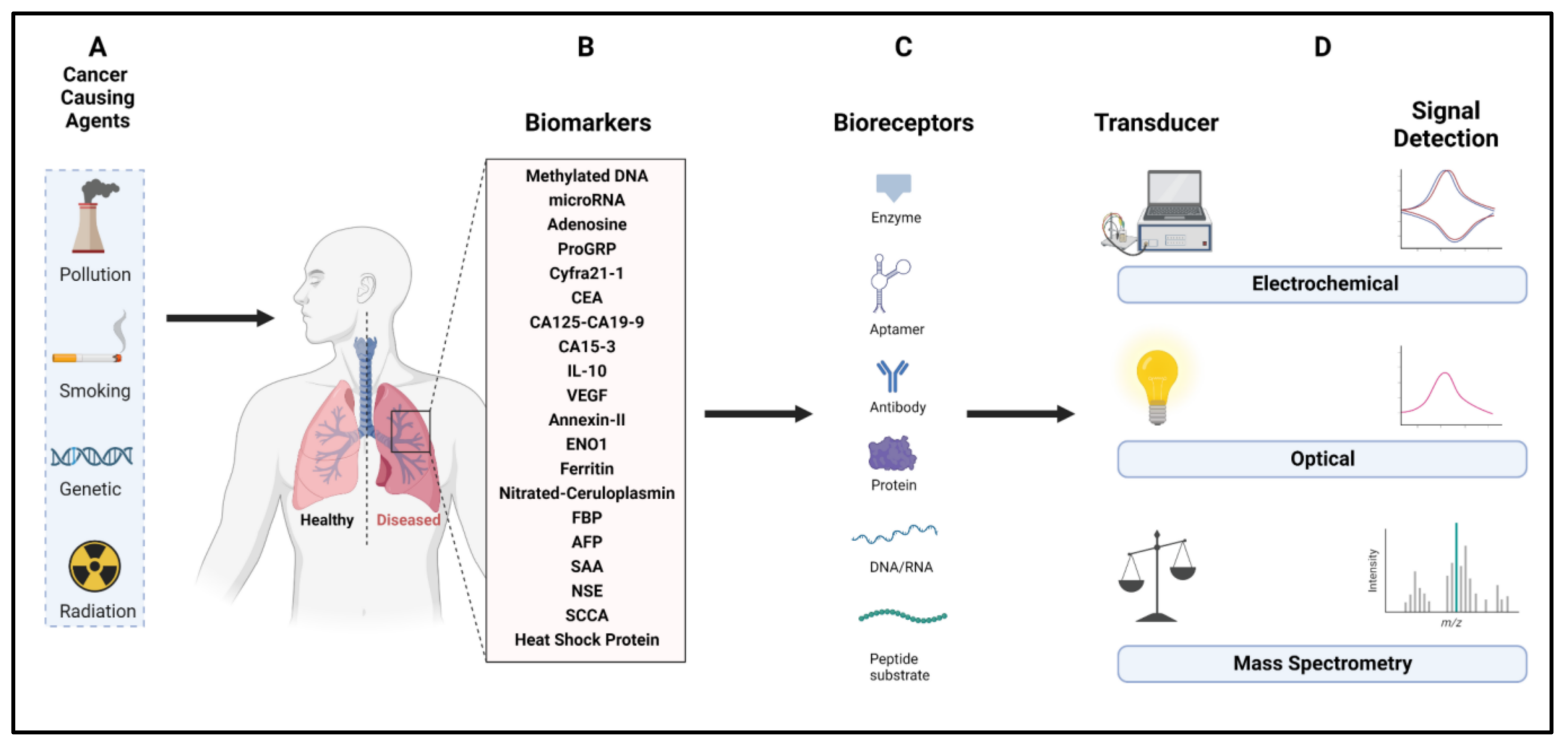
Schematic representation of lung cancer induced by various tumor-causing agents and the detection of cancer by biosensing methodologies. (**A**) Variable cancer-causing agents such as smoke, pollution, exposure to radiation, and genetic effect lead to liver cancer; as a result, many biomarkers are produced at the usual levels and released in the body fluids. (**B**) Liver-cancer-associated biomarkers including methylated DNA, microRNAs, and genes are listed. Biosensors are developed based on the recognition of biomarkers by the bioreceptors, which are integrated with the transducer and the signal amplification systems. (**C**) Different types of bioreceptors can recognize the biomarkers and transfer the signal to a (**D**) signal amplifier that amplifies the signal to a readable format (generated from biorenders.com, accessed on 12 June 2023).

Biosensors are generally defined as self-contained, highly sensitive, small bioanalytical devices that combine biological recognition molecules such as enzymes, antibodies, and nucleic acids with physicochemical transducers and detectors to convert recognition signals into detectable output signals to detect interacting analytes [16,18]. Biosensor components include an electronic system consisting of a bioreceptor/biorecognition element (BRE), a transducer, a processor, and a display. A bioreceptor is a molecule that recognizes an analyte. Transducers convert one form of energy into another. The transducer produces a quantifiable signal corresponding to the presence of the target analyte present in the specimen as depicted in Scheme 1.

The majority of transducers generate optical or electrical signals, which are typically proportionate to the number of interactions between the analyte and bioreceptor. The electronic system is responsible for processing the transduced signal so that it can be displayed. It consists of an intricate electrical circuitry that amplifies and converts impulses into digital signals. The biosensor’s display device then quantifies the signals that have been processed. Depending on the needs of the user, the output signal on the display may be numerical, visual, tabular, or even a picture [19]. Biosensors can be divided into several groups based on the biotransducer [20]. A few common categories of biotransducers include electrochemical, optical, thermal, and piezoelectric biosensors. Aptamer-based lateral flow assays are low-cost, rapid, and easy to use in point-of-care testing. This self-health monitoring would be a ultimate method to follow up on personal health periodically [21]. To enhance the sensitivity of the sensors, nanomaterials have been used [22]. AuNPs/conducting polymer nanocomposites were used for the fabrication of amperometric nanobiosensors for the ultrasensitive detection of non-small-cell lung cancer [23]. Applications of biosensors include illness monitoring, drug screening, and detection of biomolecules that are either disease markers or therapeutic targets. Electrochemical biosensing methods, for example, can be utilized as clinical tools to identify protein cancer biomarkers [19].

## 2. Overview of Lung Cancer

Neoplasia is a disease process characterized by the aberrant growth of human cells. Despite their similarity to normal cells, there are expected to be important differences in protein expression patterns, expression levels, cell-type-specific gene expression patterns, etc., as part of the disease process. These differences can be exploited to detect biomarkers. Furthermore, since these biomarkers may appear before tumors grow, they can help screen for preneoplastic growth, allowing for accurate monitoring, early diagnosis, and personalized treatment [12]. In the case of lung cancer, this can help in making proactive clinical decisions, therapeutic course corrections, drug dose attenuations, and patient stratifications for clinical investigations. In lung cancer, one gene that is not only highly expressed within lung cancers but is also expressed within pre-neoplastic lesions is the epidermal growth factor receptor (EGFR). EGFR is a tyrosine kinase receptor involved in processes related to proliferation, metastasis, and apoptosis in cancerous lesions. EGFR has been shown to increase in expression in the early steps of carcinoma development and is associated with poor prognosis [10,11,24]. Additionally, it may exhibit mutations in the kinase portion, which may amplify its antiapoptotic effects. Therefore, accurate and disease-relevant diagnostic methods may leverage the detection of EGFR levels in cells to identify carcinomas in their early stages. Similarly, Ki-67 also shows increased expression in the early stages of carcinoma, which may help predict prognosis [25]. Changes in genetic sequence can be identified through nucleic acid amplification assays (NAATs), which can target specific portions of the genome and amplify their number, which allows for the detection of various mutations (Epidermal growth factor receptor, Tumor protein p53, Anaplastic lymphoma kinase and Phosphatidylinositol-4,5-bisphosphate 3-kinase catalytic subunit alpha) within the gene [26].

Similarly, various proteins involved in controlling the cell cycle may also show changes in their structure and function, which can have a heavy impact on cell proliferation, apoptosis, and differentiation [26,27]. These changes may occur at the genomic level, but in some cases may also occur after transcription (as in alternative splicing), or post-translation (as in methylation). These changes can be detected by biosensing strategies.

## 3. Biomarkers and Their Biosensors

A biomarker or a biological marker is an objective indicator of normal physiology or pathophysiology and may be evaluated as an indicator of pharmacological response to therapeutic interventions. Broadly speaking, it is an umbrella term that incorporates measurements of any parameter that poses relevance to a given clinical scenario [28]. Biomarkers are useful for early diagnosis of lung cancer and include the following.

### 3.1. DNA Methylation

Lung cancer can be caused by a combination of genetic and epigenetic alterations, including DNA methylation and accumulation. DNA methylation is the result of the enzymatic addition of a methyl group at the fifth carbon of the cytosine ring (5mC) [29]. It does not alter the DNA sequence; however, 5mC distribution across the genome regulates genomic imprinting and inactivation of the X-chromosome [30,31]. DNA methylation is also important to maintain genomic stability and prevent somatic mutations [31,32]. The demethylation pathway to remove the methyl group from 5mC facilitates the cells to regulate the level of 5mC and control the gene expression and protein turnover based on their requirements. The information encoded by the methylated sequences can be useful for tracing out this unique cell and its function, which can be an important tracking method for the onset of many diseases, including cancers [33,34]. The correlation between lung cancer and DNA methylation leads to much interest as it is a promising biomarker for lung cancer diagnosis, prognosis, and treatment [35]. Different kinds of approaches have been applied to quantify DNA methylation levels for clinical and research purposes [36,37]. Bisulfite conversion, which converts C into U (5mC) in unmethylated genes, leaving methylated genes unchanged, is widely used for the analysis of DNA methylation. Additionally, methylation-specific polymerase chain reaction (MS-PCR), methylation-sensitive single nucleotide primer extension (MS-snuPE), methyl light, and melting curve analysis combined with MS-PCR can also quantify DNA methylation [38]. For accurate quantification of 5mC levels from bisulfite conversion, microarray techniques are integrated [39]. Alternatively, enzyme-linked immunosorbent assay (ELISA) based methylation assays are used to avoid bisulfite conversion. Yet, this method is less sensitive, and external controls are used for the quantitative analysis. Methyl-binding domains (MBD, proteins, or antibodies) are used for specific methylated sites in a DNA sequence [40]. In addition, high-performance liquid chromatography (HPLC) and mass spectroscopy (MS) have been used for the quantification of DNA methylation; however, these methods need a large number of samples.

Recently, biosensors have been developed by integrating the different analytical methods used for the accurate and specific detection of methylated DNA. For example, in Forster resonance energy transfer (FRET), ligase chain reaction, hybridization chain reaction, paired-end tagging, and super sandwich DNA structure assembly, the split fluorophore and nanomaterials strategies are used to enhance the performance of methylated DNA detection.

Among the biosensing techniques, electrochemical sensing strategies are considered to be more advantageous due to their miniature form and low cost. Exonuclease III can be used to construct a target recycle-based signal amplification under isothermal conditions, which is an alternative to sophisticated PCR amplification [41]. Target-induced conformational change in the probing DNA mechanism has been used to detect methylated DNA by an electrochemical method using exonuclease as a target recycler. Both ends of the probe DNA in a stem–loop structure were labeled with thiol and methylene blue, and the thiol groups were attached to Au nanoparticles. In the presence of auxiliary DNA, the DNA probe formed a double-stranded DNA duplex, exhibiting low current, and the 5mC remained unchanged after bisulfite treatment. When methylated DNA was added, it formed a complete duplex with the DNA probe, but the wild-type DNA mismatched with the auxiliary DNA; Exonuclease III digested the auxiliary DNA, leaving the methylated DNA to form a duplex with the next available auxiliary DNA. Thus, the methylated DNA amplified the signal. However, exonuclease III did not alter the wild-type duplex. The LOD of this method was found to be 4 fM [42].

**Figure 1:**
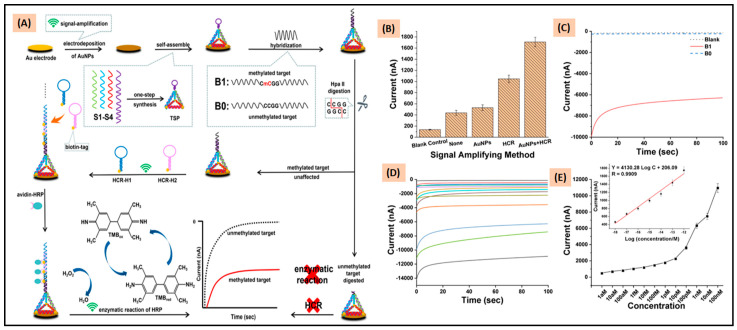
(**A**) Schematic representation of methylated DNA detection by multiple signal amplification system for biosensing application. (**B**) Verification of signal amplification from the fabricated biosensor. (**C**) Change in the chronoamperometric signals for control (dot), unmethylated target (dash), and methylated target (solid); (**D**) variation in the chronoamperometric signals in the presence of methylated DNA in the range of 0 aM to 100 M (top to bottom). (**E**) The dose-dependent variation in the signal with different target concentrations. A linear relationship between the chronoamperometric signals and the logarithm of target concentrations in the range of 1 aM to 1 pM is shown in the insert. R represents the correlation coefficient and the error bars were obtained from more than one replicate. Adapted from [47], with copyright permission under the terms of the CC-BY-NC-ND 3.0 license.

The P53 tumor suppressor gene, the most frequently methylated gene in the human genome, plays a major role in carcinogenic processes [43]. When its CpG island, in the promoter region, is unusually methylated, downstream genes are sliced steadily, which is a major cause of cancer. To detect p53 methylation, an electrochemical sensor that uses bisulfite conversion was developed. Peptide nucleic acid (PNA) was used as the capture element and [Ru(NH_3_)_6_]^3+^ was used as an electroactive material. The p53 gene was first treated with bisulfite conversion, which changes the Watson–Crick base-pair behavior of methylated and unmethylated p53 genes, allowing the complementary DNA probe to easily capture methylated genes by hybridization. The unmethylated genes, however, are not recognized by the DNA probe and therefore are not captured. This method can detect the methylated p53 gene at concentrations as low as 18 pM [44]. In another electrochemical study, sandwich electrochemical genosensors were designed for the detection of gene-specific methylation using Fe_3_O_4_/N-trimethyl chitosan/gold (Fe_3_O_4_/TMC/Au) nanocomposites for tagging the DNA probe and polythiophene as the sensing element sensing platform. The detection limit of this method under optimal conditions was 2 fM [45]. Zhou et al. constructed a ratiometric electrochemical biosensor for the sensitive detection of methylated DNA with a multi-step DNA amplification circuit. The proposed method is highly sensitive and can detect concentrations as low as 4 aM [46]. Tetrahedral DNA-based capture probes are often used to detect methylated DNA as they can easily find the target and exhibit minimal non-specific adsorption, ordered orientation, and controlled spacing. Chen et al. constructed a stem–loop, tetrahedron, composite DNA-capturing probe attached to an Au nanoparticle-coated gold electrode. This design consisted of a restriction enzyme digestion of HpaII, signal amplification, electrodeposition of Au nanoparticles, hybridization chain reactions, and horseradish peroxidase enzymatic catalysis. The sensor showed a LOD of 0.93 aM [47]. The design, construction, and results of the biosensor are shown in Figure 1. A different sensor using padlock-probe, primer-generating, rolling circle amplification (RCA) was used for the ultra-sensitive electrochemical detection of methylated DNA. A linear padlock, circulated after bisulfite treatment of methylated DNA, served as a template containing a DNA tetrahedron for RCA. The DNA tetrahedron immobilized onto a gold electrode was used as a nanocarrier. This method achieved a LOD of 0.1 aM [48]. A graphene oxide (GO)-based sensing platform has also been explored for the detection of methylated DNA. Anti-5-methylcytosine antibody was immobilized with GO, which bound to CpG methylation sites with high specificity. An IgG secondary antibody labeled with horseradish peroxidase (HRP-IgG) later bound to it. In the presence of H_2_O_2,_ the HPR-IgG oxidized the hydroquinone into benzoquinone, which caused electrochemical signal changes. The concentration of methylated DNA was directly proportional to the change in the electrochemical signal, which led to the quantification of the methylated DNA at concentrations as low as 1 fM [49]. 

**Figure 2:**
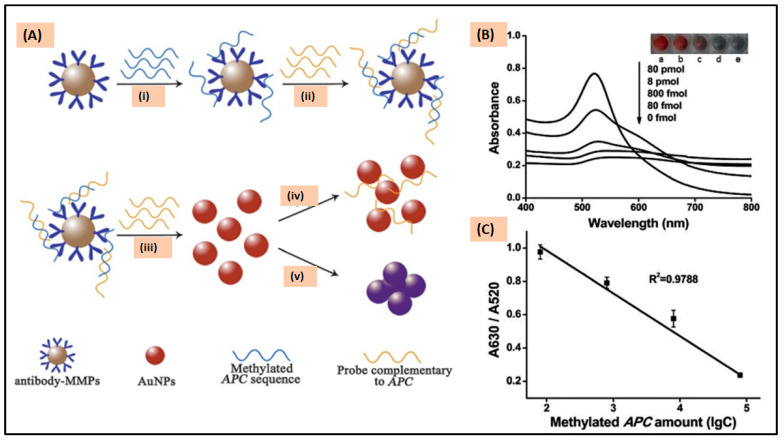
(**A**) A pictorial representation of methylated adenomatous polyposis coli (APC) sequence detection mechanism: (i) in the first step, methylated APC target sequence was captured by immobilized MMPs; (ii) a partial complementary to the target probe was introduced to form partial dsDNA duplex; (iii) the captured probe dissociated from the hybrid-conjugated microspheres by heat denaturation; (iv) AuNp was used as a probe to detect the released target sequences. There is no significant change in the solution (red) after NaCl was added. (v) Since there are no methylated APC target sequences, and the functionalized MMPs did not capture the unmethylated APC and there was no duplex formation, therefore, the color of the solution changed from red to purple rapidly. (**B**) Colorimetric detection of methylated APC sequence and the change in the absorption spectra of AuNps with different concentrations of APC in the range of 80 fM to 80 pM. (**C**) Calibration plot of absorbance ratio against the target. The error bar indicates the standard deviation of the results from four different experiments. Adopted from [51], with copyright permission under the terms of the CC-BY-NC-ND 3.0 license.

Colorimetric biosensors for the detection of methylated DNA are more advantageous due to the direct observation of color changes by the naked eye, a rapid response, and a low cost. Gold nanoparticle aggregation and HRP-based colorimetric assays are often used for the detection of methylated DNA. Su et al. developed a ligase chain reaction (LCR) that, when integrated, increased the sensitivity of the method. LCR facilitated the exponential amplification of the signal through ligase-assisted cycles of DNA ligation, which made its sensitivity comparable to that of PCR. One of the probes was modified with phosphorothioate, and the dsDNA produced from the LCR remained unchanged after treatment with ExoI and ExoIII. The color change from red to blue distinguished methylated from unmethylated DNA without LCR reaction [50]. Another AuNps-based colorimetric assay used magnetic microspheres (MMPs), conjugated with an anti-5-methylcytosine monoclonal antibody, to capture the methylated CpG target. A partially complementary DNA sequence, by contrast, formed a weak dsDNA duplex. The antibody-conjugated microspheres were then magnetically separated and incubated at high temperatures to release the captured DNA probes into the solution. Finally, the DNA was quantified by the AuNp-aggregation colorimetric method. The ssDNA probes were adsorbed on the AuNp surface by electrostatic forces, preventing AuNp aggregation in the presence of NaCl. The probes were then released proportionally to methylated DNA and could be detected with high specificity at concentrations as low as 80 fM. On the other hand, as unmethylated DNA does not have 5mC, it was not captured by the antibody, and the probe was not released into the solution. Therefore, AuNp instantly aggregated and changed from red to purple, which was visually observed as represented in Figure 2 [51]. Chen et al. also developed a simple colorimetric method for the detection of methylated DNA. AuNp was conjugated with ssDNA complementary to the P-16 gene. Then, methylated and unmethylated P-16 genes were treated with bisulfite, PCR amplified, and finally incubated with AuNp-conjugated probes in a NaCl solution. The probes then paired with the amplified, methylated DNA, inducing AuNp aggregation. Meanwhile, unmethylated P-16 genes do not amplify after bisulfite treatment and therefore did not pair with the ssDNA probe, leading to no aggregation or color change [52]. Methylene blue, a derivative of phenothiazine, undergoes optical changes upon interaction with DNA. An ssDNA complementary probe was hybridized with the methylated and unmethylated DNA. Methylene blue was intercalated with both types of dsDNA duplexes and its optical properties were observed. Compared to unmethylated DNA, both the absorbance and fluorescence of methylene blue were significantly reduced when treated with the methylated DNA-probe duplex [53]. A methyl-binding domain (MBD) colorimetric assay was used to detect methylated DNA, at low inputs of 50 ng or less, within 2 h. The biotin-labeled methylated DNA was recognized by the streptavidin-conjugated horse radish peroxidase (SA-HRP) by biotin–streptavidin binding interactions. The degree of methylation was visually displayed by HRP-mediated reduction of 3,3′,5,5′-Tetramethylbenzidine (TMB). The color intensity is directly proportional to the amount of methylated DNA [54].

**Figure 3:**
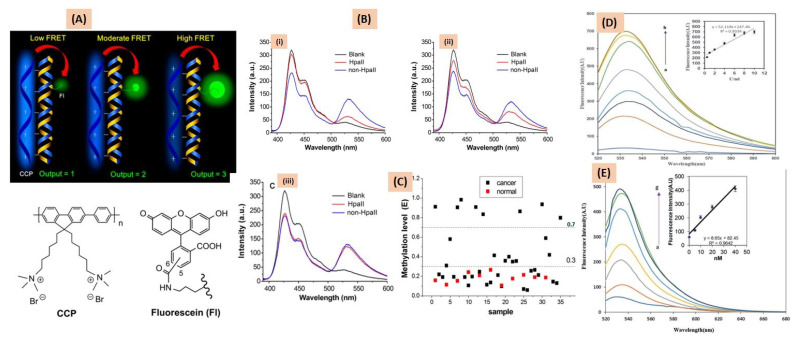
(**A**) Determination of methylated levels of cancer-related genes from the CCP-fluorescein FRET pair. The chemical structures of the donor CCP polymer and the acceptor fluorescein are represented. (**B**) The FRET efficiency in the presence of different levels of methylated DNA: (i) low level of methylated DNA, (ii) moderate level of methylated DNA, and (iii) high level of methylated DNA. Fluorescence emission spectra were recorded from a Hitachi F-4500 fluorometer exciting the samples at 380 nm. (**C**) The level of methylation in 35 RASSF1A promoter cancer samples and 11 healthy samples were analyzed from the CCP-based FRET technique. Adapted from ref. [58], with copyright permission under the terms of the CC-BY-NC-ND 3.0 license. (**D**) The methylation levels of FAM-oligo-Au DNA duplex are indicated from the fluorescence spectra of (1 mg/mL) upon enzymatic reaction of a series of concentrations of M.SssI MTase enzyme (a–h: 0, 0.5, 1, 2, 4, 6, 8, 10 U mL). The insert represents the linear relationship of fluorescence intensity against M.SssI MTase enzyme concentration. (**E**) Change in the fluorescence signal of (1 mg/mL) FAM-oligo-Au ssDNA probe duplexed with variable concentrations of methylated DNA targets (a–g: 0 pM, 5 pM, 10 pM, 20 pM, 40 pM, 70 pM, 100 pM). The insert represents the linear relationship of fluorescence intensity against methylated DNA concentration. Adapted from [55], with copyright permission under the terms of the CC-BY-NC-ND 3.0 license.

Fluorescence-based detection of methylated DNA was designed by Karimi et al. In this platform, DNA labeled with FAM was bound to AuNps by Au-S bonds, FRET was generated by bringing FAM and AuNps into close proximity, and methylated DNA was detected by fluorescence. Hybridization of the complementary probe followed by methyl transferase (M. Tase) enzyme activity introduced methylation in the DNA duplex. The additional methyl group physically separated FAM and AuNps, which resulted in the recovery of the quenched fluorescence [55]. Similar results were exhibited in the presence of methylated complementary DNA sequences. An increase in the fluorescence intensity was directly proportional to the concentration of the methylated DNA present in the sample. The sensitivity of this method was 2.2 pM, Figure 3 [55]. In another fluorescence-based study, Hori et al. designed and synthesized a molecule–protein hybrid probe, integrating a DNA-binding fluorophore with a methylation-binding domain. When this probe interacted with methylated DNA, the fluorescence intensity increased, allowing the monitoring of methylated DNA during mitosis [56].

Donor–acceptor fluorophore-inducing cationic conjugated polymers (CCPs) form a CCP-ssDNA complex as the result of electrostatic forces between positively charged CCP and negatively charged DNA [57]. CCP is highly fluorescent in aqueous media, with absorption and emission maxima of 380 nm and 424 nm, respectively. The emission spectra of CCP overlap with the absorption spectra of fluorescein, which is the basic requirement to perform FRET analysis. Fluorescein-labeled DNA can be detected by the CCP-based FRET mechanism. Zhang et al. designed a FRET-based assay for the detection of methylated DNA based on this concept. In this study, methylated and unmethylated DNAs were digested by endonuclease HpaII restriction enzyme, which cleaved the 5′-CCGG-3′ site of the unmethylated DNA, while methylated DNA remained unchanged. After digestion, methylated DNA acted as a template for PCR amplification using Fl-dNTPs. However, digested, unmethylated DNA had no PCR amplification. After CCP-PCR product interaction, FRET was observed only in CCP to fluorescein in methylated DNA. The degree of methylation in RASSF1A, OPCML, and HOXA9 promoters of 35 ovarian cancer samples was evaluated as illustrated in Figure 3 [58].

Cytosine methylation sites can be specifically detected using the FRET mechanism between upconversion nanoparticles (UCNPs) and gold nanorods (AuNRs) with the aid of methylation-sensitive HpaII and methylation-insensitive endonuclease restriction enzymes. These enzymes recognize double-stranded 5′-CCGG-3′·5′-CCGG-3′. In this mechanism, a probe DNA with a methyl group is coupled with UCNP and AuNR at both ends by Au-S bond and streptavidin–biotin affinity interactions, respectively. When partial complementary sequences with and without methylation were incubated with the probe, in the absence of restriction enzymes, both duplexes showed FRET. A duplex with an hemimethylated DNA target was treated with HpaII, and no more FRET was observed between UCNP to AuNR due to the clevage of dsDNA at the restriction site. In contrast, duplexes targeting methylated complementary DNA were unaffected by HpaII and showed no change in FRET. However, incubation with the methylation-insensitive MspI enzyme resulted in the cleavage of duplexes and limited FRET. Thus, the level of CpG methylation in a particular DNA sequence can be determined by the relative fluorescence of UCNPs after treatment with HpaII and MspI. This method can detect concentrations as low as 7 pM [59].

A fluorometric nanobiosensor was developed for the label-free detection of methylated DNA using graphene quantum dots (GQD). GQD intercalated into the major groove of double-strand DNA with a high affinity. The fluorescence intensity of this nanosensor was quenched when it interacted with methylated DNA, whereas the fluorescence signal increased with unmethylated DNA, with an LOD of 73 pM. The two different behaviors of GQD in the presence of methylated and unmethylated DNAs are assumed to be changed in the conformation of dsDNA caused by the additional methyl group in the DNA. The additional methyl group altered the GQD intercalation mechanism, which in turn affected the fluorescence behavior of the nanobiosensor [60]. The fluorescence behavior of DNA-intercalating dye was used for the sensitive detection of DNA methylation.

Ouyang et al. designed a label-free fluorometric nanosensor with the interaction of DNA-intercalating dye, methylation-sensitive restriction endonuclease, and carbon nanomaterials. In these sensors, ssDNA adsorbed onto the carbon nanomaterial by π-π stacking interactions while dsDNA did not. The fluorescence of intercalating dye, SYBR green-1, was quenched when adsorbed on the carbon nanomaterials. In the presence of unmethylated dsDNA, SYBR green-1 intercalated into the major groove of the duplex, which led to an increase in fluorescence. With methylated dsDNA, the duplex was cleaved by methylation-sensitive restriction endonuclease, allowing free dye molecules to adsorb, losing their fluorescence. Unmethylated dsDNA was unaffected by the methylation-sensitive restriction endonuclease. This method can detect concentrations as low as 73 pM [61].

Capped CdTe quantum dots can be used as a fluorescence probe for the detection of methylated DNA. The probing quantum dot intercalated with unmethylated dsDNA, enhancing the fluorescence signal, while no noticeable change in the fluorescence intensity was observed in the presence of methylated DNA. This observation was further confirmed by different mobility electrophoresis assays. The LOD of this method was reported as 62 pM [62]. Several other methods such as surface plasmon resonance (SPR), surface-enhanced Raman scattering (SERS), and microfluidic systems have been thoroughly reviewed as highly sensitive methods to detect methylated DNA [63].

### 3.2. MicroRNA

It is well documented that miRs play a significant role in lung cancer development and progression, and circulating miRs have already been used as biomarkers for non-small-cell lung cancer [64]. Lung cancer patients and healthy individuals can be differentiated by aberrant circulating miR levels [65]. They are associated with different features of cancer cells, such as cell growth, apoptosis, metabolism, and invasion [66]. MiRs are short non-coding RNAs with lengths ranging from 18 to 24 nucleotides. MiRs inhibit the translation of messenger RNA (mRNA) and degrade mRNA by base pairing with the complementary sites of target mRNA. MiRs control gene expression by this mechanism at the post-transcriptional stage. The development of a miR detection method is difficult because they are short in length, are present in body fluids in trace amounts (0.01% of total RNA mass), and have different secondary structures and nucleotide composition. In addition, many miRs are very close to each other, with single-nucleotide differences, making it a major challenge to design detection methods with high sensitivity and specificity. Despite all this, it is important to identify miR biomarkers specific to a low-cost diagnostic technique. Many analytical techniques including Northern blotting, in situ hybridization, microarrays, and nucleic acid amplification-based methods have been used for miR detection [67,68]. However, they are not sensitive enough for the detection of a low abundance of miR. Though Northern blot is the standard method for miR quantification, it is laborious and requires radio labeling [69]. Real-time quantitative polymerase chain reaction (RT-qPCR) is the standard method for the quantification of miR due to good sensitivity and accuracy. However, it cannot be used in point-of-care settings due to its high cost and the need for trained professionals.

Fluorescence-based assays for the sensitive detection of miR by competitive DNA displacement without amplification have been developed. In these methods, FAM-labeled cDNA of miR and a short DNA strand labeled with a fluorescence quencher form a dsDNA duplex. Due to the proximity of the FAM and quencher, the FAM’s fluorescence is significantly reduced. When the target miR is introduced, the short quencher-labeled DNA is displaced by the miR, forming a FAM-cDNA-miR duplex, increasing the fluorescence signal. This method can detect miR at concentrations as low as 1 pM [70]. A duplex-specific nuclease-assisted CRISPR-Cas12a strategy has also been reported to detect miR using a personal glucometer. In this assay, the target miR is partially hybridized with complementary DNA. The duplex-specific nuclease cleaves the DNA in the RNA-DNA duplex, releasing the rest of the ssDNA. MiR further reacts, generating a large number of DNA strands. The amplified DNA activates the collateral cleavage activity of CRISPRCas12a, which links sucrase to the surface of magnetic beads (MBs). The sucrase is then released, allowing it to convert sucrose into glucose, which is measured using the personal glucose meter. MiR21 and miR-205 were detected at LODs of 2.4 and 1.1 pM, respectively. This highly sensitive strategy, with low interference from other components, could be promising for miR detection in point-of-care testing [71]. Digital flow cytometry-ligation rolling circle amplification (dFC-LRCA) has been used for multiplexed detection of miRs in a homogeneous solution. Bacteria-sized RCA nono-flower balls (NFBs) were produced by target miRs through LRCA. The fluorescent oligos were hybridized with NFBs and counted directly using digital flow cytometry. Three different miRs were detected using this method, and the respective LODs of miR, miR-141, and Let-7a were 3.09 pM, 1.58 pM, and 1.34 pM, respectively [72]. Recently, diverse analytical methods for multiplex detection of different kinds of miRNAs were reviewed [73]. Fang et al. developed a different type of fluorometric detector, using two complementary peptide nucleic acids (PNAs) for base pairing that are labeled with dicysteine units at the terminals. The short PNA complementary sequence is linked more strongly than natural nucleic acids. After hybridization, the two Cys-Cys units were in close proximity, forming a split tetra-cysteine motif, and were complexed with bis-arsenite dyes, such as FlAsH or ReAsH, across the nick site. The resultant complex showed high fluorescence. Changes in the fluorescence signal were directly correlated to the quantity of miR in the sample [74]. Three different fluorophore-labeled molecular beacons for the quantative detection of miR-21, miR-375, and miR-27 have been used for the simultaneous multiplex detection of miR targets present in exosomes from breast cancer cells. The stem–loop structured molecular beacons were labeled as FAM, Cy3, and Cy5 and the respective quenchers on the other ends, and the fluorescence was quenched in the absence of the target miRs. These dyes emit different wavelengths and can be used to quantify miR-21, miR-375, and miR-27. The loop sequence was complementary to the miRs, and in the presence of the target, the stem–loop structure opened and formed a dsDNA-RNA duplex. As a result, the fluorescence increased, which was correlated with the quantity of the miRs present in the sample [75]. Colorectal cancer exosome biomarker was isolated using anti-CD-63 aptamer, a capturing element from the lab-on-a-chip microfluidic platform. In this method, anti-CD-63 was conjugated on the surface of magnetic nanobeads and the isolation of the exosome was achieved by applying a transverse magnetic field along with a microfluidic channel. The channel was exposed to alternate trapping and releasing. The carbon-nanomaterial-coated magnetic beads were used as a fluorescence quenching platform, which quenches the fluorescence of the labeled aptamer in the presence of aptamer; however, in the presence of aptamer, it detaches from the nanomaterial, binds to the CD-63 target protein, and enhances the fluorescence signal. This method can detect concentrations as low as 1457 particles /mL [76].

Electrochemical methods are also widely used for the detection of miRs using electroactive molecules as redox signal reporters. Jou et al. designed an electrochemical sensor for the detection of miRs using a AuNps modified screen-printed carbon electrode (SPCE) immobilized with a hairpin DNA probe. The redox molecule and methylene blue were labeled at the 5′-end of the hairpin DNA probe. In the absence of the target miR, the MBs were near the electrode surface, resulting in efficient electron transfer between the MBs and the electrode, and a high response was noticed due to MB oxidation. Meanwhile, in the presence of target miR, the hairpin was found to be opened through displacement amplification and duplex-specific nuclease reactions. This hairpin-to-duplex transition moved the MBs away from the electrode surface, and the MB oxidation signal decreased. This method can detect miR-155 at concentrations as low as 3.57 fM [77]. Another study reported the multiplex detection of three different miRs using reduced graphene oxide/poly(2-aminobenzylamine)/gold nanoparticle-modified electrodes. On the electrode surface, a porous hollow silver–gold nanoparticle (PHSGNP) was tagged with different metal ions to enhance the electrochemical signals. The surfaces of the PHSGNs were further coupled with different capturing DNAs (cDNA) complementary to target miRs. The anti-deoxyribonucleic acid (DNA)- miR duplex antibody S9.6 was used to detect the multiple miRs simultaneously. The electrochemical sensor showed high selectivity, stability, and sensitivity with LODs of 0.98 fM, 3.58 fM, and 0.25 fM for miR-155, miR-21, and miR-16, respectively [78]. To understand the electrochemical biosensors for the miR cancer biomarkers and their limitations, the readers can refer to more specialized reviews [79].

Paper-based lateral flow assay (LFA) is one of the most favorable methods for detecting the target molecule in POC testing. The results of the assay can be observed visually. Colorimetric and fluorescent LFAs have been developed using gold, silver, and selenium nanoparticles (NPs), QDs, upconversion nanoparticles, and fluorescent dyes as detection probes. There are several methods for the detection of mat targets using LFA, but only a few reports are available on the detection of miRs. Recently, AuNp-based LFAs were developed for the visual detection of DNA. In this method, capture DNAs were used to recognize the target miRs, followed by different colorimetric strategies for visual detection [80,81]. Another study by Feng et al. reported a pH-responsive miR amplification method that detected miR using a simple test paper. The target miR was amplified using the highly efficient isothermal amplification technique of netlike rolling circle amplification (NRCA). During the amplification processes, a large quantity of H^+^ was produced. This change in the pH could be monitored using pH test paper, with the color intensity indicating the amount of miR present in the sample [82]. It is a very simple, rapid, and low-cost method that can be used for point-of-care applications.

### 3.3. Adenosine

Adenosine is a nucleoside molecule that has been well studied as a biomarker for lung cancer. Adenosine levels have been found to be elevated in lung cancer patients [83,84]. Adenosine is thought to accumulate due to cell necrosis caused by hypoxia and rapid growth of cancer cells. Typically, adenosine is excreted renally [85,86,87]. Biosensors, such as electrochemical and surface plasmon resonance-based detectors have been developed to measure adenosine levels with high sensitivity and specificity and have become promising candidates for early lung cancer diagnosis [87,88,89,90]. Electrochemical biosensors involve immobilizing adenosine-specific antibodies or aptamers on electrode surfaces and measure the change in electrical current that occurs upon adenosine binding [91,92]. For example, Runsewe et al. developed a conducting polymer-based electrochemical biosensor for highly sensitive adenosine detection. In this study, the authors used 3-aminopropyl triethoxysilane (APTES) and 3-thiophenecarboxylic acid (3-Th-COOH) to electrochemically polymerize EDOT and ProDOT-(COOH)_2_ to prepare an indium-tin-oxide-coated (ITO-coated) glass electrode. COOH groups on the electrode’s surface were exploited to immobilize a modified, adenosine-specific aptamer. The detection limit of the electrochemical adenosine sensor with this aptamer was 2.33 nM with a linear range of 9.6 nM to 600 µM. The sensor was highly selective, specific, stable (6 days), and could be stored in PBS or under argon.

Similarly, a competitive electrochemical sensor for the detection of adenosine was developed by Sanghavi et al. The proposed sensor showed the capability to quantitatively monitor adenosine in real time without the need for washing. The authors used an ATP-specific aptamer as a recognition receptor and a pre-complexed, electroactive, flavin–adenine dinucleotide aptamer to monitor the competitive binding of the aptamer to ATP. The electrochemical signaling surface was designed by simple modification of a carbon paste electrode with graphene and gold nanoparticles (Gr-AuNP-CPE). When ATP binds to the aptamer complex, a proportional amount of flavin–adenine dinucleotide is released. Analysis can be performed in 12 min and allows a wide working range of (1.14 × 10^–10^ to 3.0 × 10^–5^ M) with a LOD of 2.01 × 10^–11^ M [93]. Wang et al. also reported a label-free aptasensor for the sensitive detection of adenosine using the co-assembling of a thiolated aptamer, dithothreitol (DTT), and 6-mercaptohexanol (MCH) on the surface of a gold electrode (Au/aptamer-DTT/MCH). The change in interfacial electron transfer resistance (Ret) of aptasensors, using [Fe(CN)6]^3−/4−^ as redox probes, varied linearly over a range of 0.05 pM to 17 pM and an LOD of 0.02 pM. The aptasensor with combined DTT/MCH co-assembly showed an improved LOD compared to sensors made with DTT and MCH alone. This method can reduce non-specific adsorption of interfering biomolecules to the electrode surface [94].

Surface plasmon resonance (SPR) biosensors, on the other hand, use the interaction of light with metal surfaces to detect changes in refractive index. Aptamers or antibodies can be used as a recognition receptor for recognizing adenosine. The change in the refractive index of the metal surface upon binding with adenosine can be used to quantify the adenosine present in a given sample [95,96]. In an interesting study, Zaho et al. reported the detection of ATP by the surface plasmon resonance (SPR) phenomenon using an ssDNA probe. The ssDNA probe consists of an ATP aptamer in the middle to recognize ATP and five cytosines (C) at either end of the probe that recognizes Ag^+^. In the absence of ATP, the probe exists in a rigid hairpin structure due to C-Ag^+^-C interaction, resulting in a high SPR signal. In the presence of ATP, however, the hairpin structure changes in three-dimensional conformation and the SPR signal changes in proportion to the concentration of ATP in the sample. The difference in signal response with the linear working concentration range from 0.05 to 500 nM was observed with a LOD of 15 pM [97].

**Figure 4:**
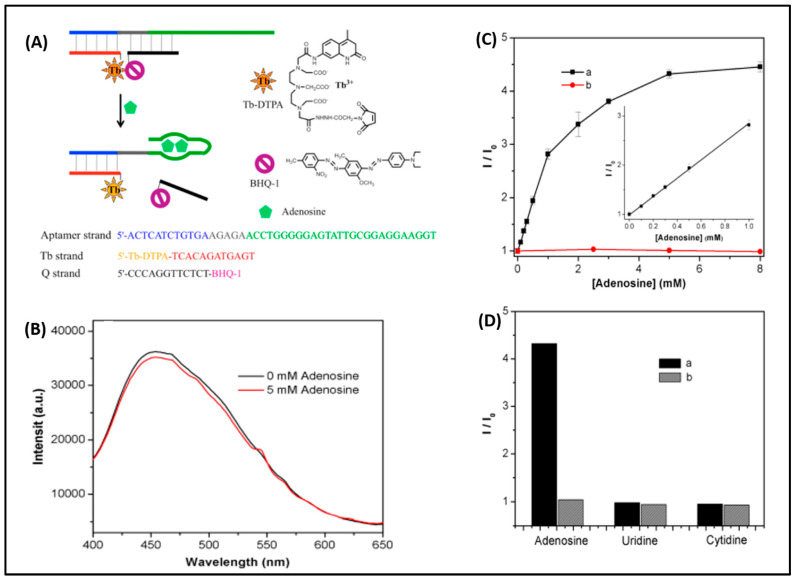
(**A**). The schematic representation of LRET between the donor and the acceptor for adenosine sensor consists of terbium complex conjugated with DNA aptamer; (**B**) steady-state emission spectra of adenosine aptasensor in the presence of adenosine in 100% serum; (**C**) change in the time-resolved (a) and steady-state (b) emission intensity of the aptasensor at 545 nm versus different concentration of adenosine in 100% serum with the delay time of 50 μs. The insert represents the linear relationship of luminescence response against adenosine concentration in the low concentration range. (**D**) The cross-reactivity of aptasenor in the presence of 5 mM nucleosides in serum based on time-resolved (a) and steady-state (b) emission measurement. Adapted from ref. [99], with copyright permission under the terms of the CC-BY-NC-ND 3.0 license.

Another strategy reported for adenosine detection is based on the luminescence phenomenon. Luminescence biosensors use the emission of light by specific chemical compounds in response to adenosine binding to a corresponding antibody on the sensor surface [98]. Li et al. reported a turn-ON luminescent aptasensor for the sensitive detection of adenosine in undiluted serum samples. Luminescence resonance energy transfer (LRET) between the terbium complex and BHQ1 quencher was utilized to detect adenosine. Three types of DNA oligonucleotides were used in this design: a Tb^3+^ chelated complex-labeled DNA, a quencher-labeled DNA, and an aptamer DNA. The aptamer DNA was extended to hybridize the Tb-labeled DNA with the quencher-labeled DNA. In the absence of adenosine, the sensor emitted a small amount of light; however, in the presence of adenosine, the DNA conformation switched, increasing emission. The LOD of this method was 60 µM [99]. The schematic representation of LRET between the donor and the acceptor is shown in Figure 4. A dose-dependent fluorescence signal and the cross-reactivity of the aptasensor with closely associated molecules are illustrated. Sun et al. reported a chemiluminescent biosensor for the sensitive and selective detection of adenosine under alkaline conditions using carbon-quantum-dot-catalyzed luminol-H_2_O_2_ system emission. The carbon quantum dots released from the surface of the aptamers activated a graphene/magnetic β-cyclodextrin polymer complex. The adenosine concentration can be measured in the range of 5 nM to 0.5 pM, with a LOD of 0.21 pM [100].

### 3.4. ProGRP

ProGRP (Pro-Glycine-Arginine-rich protein) is a primary biomarker for lung cancer commonly studied for its potential use in early diagnosis [101]. ProGRP is a product of the cleavage of larger proteins and is elevated in the serum of lung cancer patients [102]. Overall, ProGRP is a valuable tumor marker for the detection and monitoring of SCLC and a good tool for discriminating NSCLC versus SCLC. Various biosensing techniques have been developed to detect its presence in biological samples with high sensitivity and specificity [103]. These methods offer a non-invasive approach for the early diagnosis of lung cancer.

Cui et al. selected ProGRP-specific aptamers, using electrochemiluminescence, to detect ProGRP. The high-affinity truncated aptamers had a K_d_ value of 16 nM and a LOD of 17 nM [104]. Sun et al. reported an aptamer-based SPR assay for the detection of ProGRP_31-98_. The aptamer on the surface of the SPR sensor was immobilized and used as a bioreceptor. When a sample was introduced into the SPR system, the SPR signal increased due to the molecular complex of the aptamer and ProGRP. The change in the SPR signal was used to quantify the ProGRP in the sample. The LOD of the proposed system was calculated to be 15.6 nM [105]. Wang et al. reported a molecularly imprinted photoelectrochemical sensor for the sensitive detection of ProGRP lung cancer biomarkers. Under optimal conditions, the sensor specifically detected the target with a dynamic range of 0.002 ng/mL to 0.5 ng/mL with a LOD of 0.0032 ng/mL [106]. Liu et al. designed an ultrasensitive electrochemical immunosensor for ProGRP detection by modifying the electrode with 3D-rGO gold nanoparticle composite (3D-rGO@Au) substrates for efficient electrical conductivity and a large surface area to immobilize the capture antibody. The detection antibody with a SiO_2_ nanosphere forms a sandwich complex with ProGRP. The sensor exhibited an electrical response in a wide range from 1 fg/mL to 10 ng/mL with a LOD of 0.14 fg/mL. The highly stable and sensitive sensor proved to be a promising device for the early screening of lung cancer and can be used for POC testing at home or in the community [107]. Zhuo et al. reported an electrochemical immunosensor for ProGRP using nanocomposite materials. AuNPs and TiO_2_ NPs were linked through 3-aminopropyltriethoxy silane (APTES), (nano-Au/TiO_2_). Secondary antibodies, labeled with ferrocene and glucose oxidase (GOD), were conjugated to nano-Au/TiO_2_ at high loadings. A cysteine/Nafion-graphene (Cys/GS-Nf) composite membrane was prepared and AuNps were self-assembled on its surface through an Au-S bond. The capture antibody was then immobilized on the Cys/GS-Nf membrane. In the presence of ProGRP, a sandwich complex formed, which led to a change in the electrical signal. A linear relationship between ProGRP concentration and signal change was found in the range of 10 to 500 pg/mL with a minimum detection amount of 3 pg/mL [108].

### 3.5. Cytokeratin 19 Fragment 21-1 (CYFRA21-1)

CYFRA21-1 is a protein biomarker that has been studied for its potential use in the early diagnosis of lung cancer. Elevated levels of CYFRA21-1 have been found in the serum of patients with lung cancer [109]. Biosensing methods have been applied to detect CYFRA21-1 levels in patient serum samples, allowing for easy measurement. These methods provide a non-invasive approach to the early diagnosis of lung cancer [110]. Studies have shown that CYFRA21-1 has a high diagnostic accuracy for lung cancer, allowing its use as a complementary tool to conventional imaging methods. However, due to inconsistent results from several studies, further research is needed to validate the use of CYFRA21-1 as a diagnostic biomarker for lung cancer, to investigate the diagnostic potential of CYFRA21-1 in larger patient populations, and to determine its clinical utility for early diagnosis [111].

**Figure 5:**
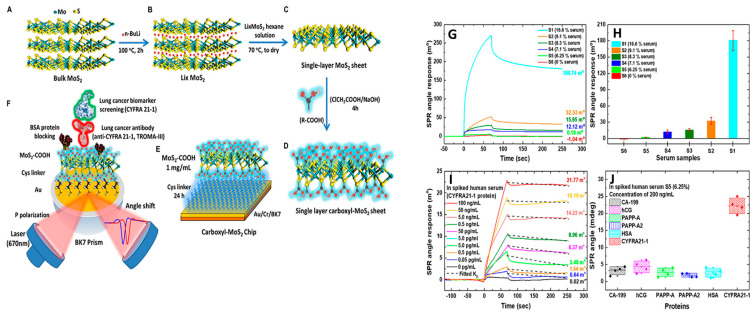
(**A**) Water-dispersible bulk MoS+; (**B**) Li intercalated MoS_2_ (LixMoS_2_); (**C**) MoS_2_ in a single layer; (**D**) single-layered carboxyl-MoS_2_ nanocomposites; (**E**) the carboxylated-MoS_2_ SPR chip; (**F**) SPR sensing mechanism for the detection of CYFRA21-1 from carboxylated-MoS_2_ SPR chip. SPR sensorgram of CYFRA21-1 spiked in human serum measured from carboxylated MoS_2_ SPR chip; (**G**) Dose-dependent sensorgram of SPR for different serum concentrations. (**H**) The SPR signal for variable serum ratios. (**I**) Quantitative detection of CYFRA21-1 protein spike in S5 buffer with different concentrations from SPR signal changes; (**J**) The specificity test of the SPR sensor with non-specific biomolecules. Adapted from [114].

Zhang et al. designed dual-mode biosensors for CYFRA21-1 based on electrochemical (EC) and photoelectrochemical (PEC) principles. This dual-signaling strategy is based on the electrochemical ratiometric strategy and the “on-off-on” PEC method. In this construction, the indium tin oxide (ITO) electrode was modified with a 3,4,9,10-perylenetetracarboxylic dianhydride (PTCDA)/C_60_ complex and gold nanoparticles (AuNPs), AuNps/PTCDA@C_60_/ITO. A double-standard DNA duplex—composed of thiol and methylene blue-labeled ssDNA, and an antibody/ferrocene (Fc)-labeled ssDNA—were conjugated, through an Au-S bond, on AuNps/PTCDA@C_60_/ITO. In the presence of CYFRA21-1, the antibody/Fc-labeled ssDNA dissociated, forming a complex with another secondary antibody conjugated to its complementary ssDNA. EC-PEC dual sensors showed a linear response in the range of 0.001–40 ng/mL with a LOD of 0.3 pg/mL for the EC and 0.0001–4 ng/mL with a LOD of 0.03 pg/mL for the PEC method [112]. Kumar et al. developed an immunoelectrode with a complex made of bovine serum albumin, anti-CYFRA-21-1, (3-aminopropyl) triethoxysilane, TiO_2_, and ITO to detect CYFRA21-1 by electrochemical impedance spectroscopy. This method showed a linear detection range of 0–12 ng/mL with a LOD of 0.24 ng/mL [113]. Chiu et al. reported highly sensitive detection of CYFRA21-1, using a carboxyl-functionalized, molybdenum disulfide (carboxyl-MoS_2_), nanocomposite-modified sensing film in a surface plasmon resonance (SPR) detection assay. The results showed a 15-fold higher affinity to CYFRA21-1 compared to the traditional SPR method. The signal response was observed in a range of 0.05 pg/mL to 100 ng/mL with a LOD of 0.05 pg/mL in the CYFRA21-1 spiked serum sample [114]. Figure 5 illustrates the preparation of MoS_2_ and sensor fabrication followed by quantitative detection of CYFRA21-1.

**Figure 6:**
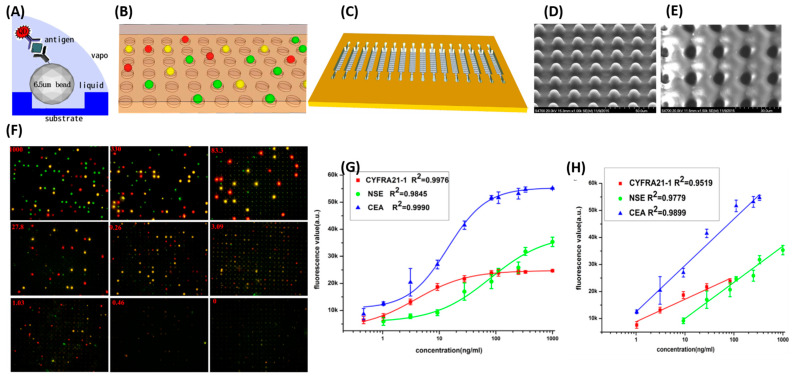
(**A**) Schematic representation of bead-based array detection method for the detection of three different lung cancer biomarkers using QDs. (**A**) Illustration of bead-based sandwich assay; (**B**) three different colored spots after sandwich complex on the chip. (**C**) The chip pattern; (**D**) SEM image of the microarray on a silicon wafer. (height = 9 μm); (**E**) SEM image of micro holes on PDMS with a diameter of 10 μm and spaced 10 μm apart from each other; (**F**) fluorescence microscopic image of the three different lung cancer biomarkers for simultaneous detection. The concentration range of antigen was 0.46–1000 ng/mL with an exposure time of 0.4 s; (**G**) the plot of fluorescence intensity against the logarithmic concentrations of CYRFA 21-1, NSE, and CEA in the multiplexed assays; (**H**) the standard calibration plot for the three cancer biomarkers. Adapted from [115], with copyright permission under the terms of the CC-BY-NC-ND 3.0 license.

A multiplex immunoassay for the detection of three different lung cancer biomarkers targets CEA, CYFRA21-1, and NSE. The construction of the sensing platform consists of the integration of suspension and a planar microarray with a single layer of polydimethylsiloxane (PDMS) made using soft lithography technology. The suspension format creates a sandwich of target protein between the two antibodies conjugated with magnetic beads and quantum dots by antigen–antibody interactions. The microarray is produced by an array of microwells in PDMS. Each microarray captures a single bead and leads to the formation of a microbead array in the chamber of PDMS. This microarray system can be used for the simultaneous detection of all three lung cancer biomarkers in 10 µL serum in the dynamic range of 1.03–111 ng/mL for CEA and CYFRA21-1 and 9.26–1000 ng/mL for NSE. The LODs for CEA, CYFRA21-1, and NSE were 0.19 ng/mL, 0.97 ng/mL, and 0.37 ng/mL respectively [115]. The image of the fabricated beads-based microarray biosensor system and the results are shown in Figure 6. Chen et al. reported an electrochemical CYFRA21-1 DNA biosensor based on a nanocomposite composed of carboxyl-functionalized graphene oxide (GO-COOH) and copper oxide nanowires (CuO NWs). Hybridization between the probe and target DNA was monitored with differential pulse voltammetry using methylene blue as a redox indicator. Under optimal conditions, the device quantified CTFRA21-1 in a linear range of 1 µM to 1 pM with an LOD of 0.118 pM [116]. Joshi et al. reported a portable and rapid electronic biosensor device based on reduced graphene oxide (rGO), melamine (MEL), antibodies, and bovine serum albumin (BSA) to quantitatively detect CYFRA21-1 in saliva. The device was tested from 1 pg/mL to 800 ng/mL of CYFRA21-1 and the corresponding response of the sensors ranged from 6.18% to 64% [117]. Jian et al. used an electrochemiluminescence (ECL) immunosensor based on an electrochemically induced atom transfer radical polymerization (eATRP) process to detect CYFRA21-1. The primary antibody was immobilized on the electrode surface and the secondary antibody was used in a sandwich assay with N-acryloyloxysuccinimide as the functional monomer. The combination of ECL and eATRP immunosensor technology demonstrated a linear relationship in the range of 1 fg/mL to 1 μg/mL with a LOD of 0.8 fg/mL [118]. Alarfaj et al. used carbon dots decorated with a ZnO nanocomposite as a fluorescent probe for the sensitive detection of CYFRA21-1 via sandwich immunoassay. BM19.21 monoclonal antibody was conjugated to a homemade carbon quantum surface. Another monoclonal antibody (KS 19.1) was physically adsorbed onto the surface of microtiter wells. In the presence of CYFRA21-1, an antibody–antigen–antibody sandwich pattern was formed and the quantity of CYFRA21-1 was monitored via changes in the fluorescence signal.

In this method, the fluorescence signal showed a linear relationship from 0.01 to 100 ng/mL with a LOD of 0.008 ng/mL [119]. For diagnostic and prognostic monitoring, Lei et al. developed a rapid quantitative detection method for CYFRA21-1 in urine via fluorescent nanosphere-based immunochromatographic test strips with europium chelate microparticles. The sensitivity of this method was 0.0071 ng/mL [120].

### 3.6. CEA

One of the typical tumor markers, carcinoembryonic antigen (CEA), is a human glycoprotein involved in cell adhesion and expressed during fetal development. CEA is overexpressed in a variety of malignancies, including colorectal, stomach, breast, ovarian, lung, and pancreatic cancers. In conventional CEA testing, immunoassay methods are often used. However, immunoassay methods require sophisticated and expensive, equipment and trained personnel. However, CEA detection can be performed via aptamer-based biosensors as well as immune-based optical and electrochemical biosensors [121], biochips [122], portable biosensors [123], mass change-based piezoelectric sensors [124], and flexural plate-wave (FPW) biosensors [125].

Wang et al. reported a photoelectrochemical (PEC) immunosensor using ascorbic acid as an electron donor that significantly changes the photocurrent density to specifically detect CEA. A heterojunction (Au@WP5/PANI-BiOBr) showed excellent PEC sensing for sensitive CEA detection. The anti-CEA antibody bound efficiently to CEA, the Au@WP5/PANI-BiOBr electrode produced relatively little current in the presence of ascorbic acid in the solution, and residual sites were blocked by bovine serum albumin. The PEC immunosensor reacted to CEA in a linear range of 0.01 ng/mL to 50 ng/mL with a LOD of 3 pg/mL [126]. Additionally, Li et al. developed a highly sensitive sandwich electrochemical immunosensor for CEA detection. A metal–organic framework (Ce-MOF) skeleton precursor was coated with hyaluronic acid (HA), then loaded with silver nanoparticles (Ag NPs) and horseradish peroxidase (HRP), and a secondary antibody (Ab2) was then attached to the surface. Ce-MoF@HA/Ag-HRP-Ab2 was used as a secondary antibody (Ab2) to catalyze hydrogen peroxide (H_2_O_2_) and signal amplification. The primary antibody (Ab1) was immobilized with AuNPs coated on the electrode. The immunosensors showed a linear response of CEA with a dynamic range of 1 pg/mL to 80 ng/mL and a LOD of 0.02 pg/mL [127]. Hwang et al. reported a magnetic-force-assisted electrochemical sandwich immunoassay (MESIA) technique for the sensitive detection of CEA from a drop of human serum using an automated point-of-care testing device. The signal response of the device showed a linear relationship in the concentration range of 0.5 ng/mL to 200 ng/mL with a LOD of 0.5 ng/mL [128]. Song et al. developed a flexible free-standing electrochemical biosensor for the sensitive detection of CEA using polypyrrole (PPy) nanocomposite film electrodes. AuNps were deposited on the film electrode and the CEA aptamers self-assembled on AuNPs by Au-S bonds. The change in electrical signals was proportional to the concentration of CEA present in the sample. The proposed sensor showed a detection capability in the linear range of 0.1 ng/mL to 1µg/mL and a LOD of 0.033 ng/mL [129]. Several electrochemical methods have been developed to detect CEA with high sensitivity, including impedance, amperometry, voltammetry, potentiometry, electrochemiluminescence (ECL), and photoelectrochemistry (PEC). A recent review of the performance of aptamer-based electrochemical sensors demonstrated outstanding LOD ranges (sub-fg/mL) for CRA [130].

Chen et al. developed an aptamer-based fluorescence assay for the sensitive detection of CEA antigens using a dsDNA duplex consisting of a CEA-specific ssDNA aptamer coupled with CuNps and its cDNA. The intercalation of CuNps was prevented in the presence of CEA and exhibited low fluorescence signal. However, in the absence of CEA, CuNps intercalated into the dsDNA duplex and emitted a strong fluorescence. This assay can detect CEA biomarkers with high accuracy over a dynamic range of 0.01–2 ng/mL with a LOD of 0.0065 ng/mL [131]. He et al. designed a label-free DNA walker biosensor for CEA detection using cascade amplification of exonuclease (Exo) III-assisted target recycling amplification (ERA). ERA generated walker DNA in the first step, followed by autonomous migration of walker DNA to substrate-modified silica microspheres in the second step. Finally, N-methylisoporphyrin IX (NMM)-assisted fluorescence signals were observed. These DNA machine biosensors could detect CEA at concentrations as low as 1.2 pg/mL with a linear range of 10 pg/mL to 100 ng/mL [132].

Mahmodi et al. reported the development of a paper-based colorimetric lateral flow assay for point-of-care detection of CEA in human serum. The results can be read by the naked eye, processed, and quantitively measured using smartphone-based analysis. Anti-CEA polyclonal and monoclonal antibodies and Polydopamine-modified AuNps were used for designing the lateral flow assay system. In the presence of CEA, sandwich immunocomplexes were formed by the antibodies with CEA at the test zone. The intensity of the red color observed was proportional to the quantity of CEA in the sample. The strips were tested in the linear range of 0.05–50 ng/mL and a visual LOD of 0.05 ng/mL with an assay time of 15 min. The results were compared with the standard ELISA method. This strip-based assay can be useful in limited laboratory resource settings for rapid and low-cost analysis and diagnosis [133]. Springer et al. reported a biofunctionalized AuNPs-assisted SPR immunosensor for the detection of CEA from blood plasma. The authors demonstrated that the modified SPR method is 1000-fold more sensitive than conventional SPR methods. This immunosensor can detect CEA in plasma down to 0.1 ng/mL [134].

### 3.7. CA15-3, CA125, CA19-9

CA15-3, CA125, and CA19-9 are biomarkers associated with lung cancer [135]. They are proteins found in the blood and can be used to monitor disease progression and response to treatment [136]. CA15-3 is a cancer antigen that is often used as a marker for breast cancer but has also been found in lung cancer patients. Elevated levels of CA15-3 in the blood can indicate the presence of cancer. CA125 is a cancer antigen that is commonly used as a marker for ovarian cancer. However, it has also been found in lung cancer patients and can be used as an indicator of the presence of the disease [136]. In order to identify a monosialoganglioside present in individuals with gastrointestinal adenocarcinoma, the monoclonal antibody carbohydrate antigen 19-9 (CA19-9) has been developed against a colon cancer cell line. It has been suggested that it can distinguish between benign and malignant pancreatic illness because it is raised in cases of gastric cancer, lung cancer, colon cancer, and pancreatic cancer [137].

Detection methods for these biomarkers include immunoassays (ELISA) and radioimmunoassays [135]. A study by Ghosh et al. concluded that among the selected indices, the combined assessment of CEA, CA19-9, and CA125 in BAL fluid and CA15-3 in the blood can be helpful in the diagnosis of lung cancer. These may be helpful in patients whose tumors are difficult to see with bronchofibroscopy or to rule out false positives. It is necessary to confirm these findings in broader populations [137].

Rebelo et el. developed a rapid low-cost immunosensor for the detection of cancer antigen 15-3 (CA-15-3) using disposable gold screen-printed electrodes (AuSPE) for point-of-care application. Anti-CA-15-3 antibody was immobilized with the COOH group of mercaptosuccinic acid self-assembled on AuSPE surface. The electrical signal change was proportional to the quantity of CA15-3 in the sample. A linear relationship with the concentration range of 1.0–1000 U/mL was observed with a LOD of 0.95 U/mL. The sensor did not cross-react with other cancer antigens, such as CA12-5 and CA19-9. Artificial serum samples were successfully analyzed using this immunosensor [138]. Wei et al. developed an electrochemical immunosensor for the sensitive detection of CA19-9 using a hybrid self-assembled membrane modified with a gold electrode. The immunosensor responded to CA19-9 in a linear range of 0.05–500 U/mL with a lower detection limit of 0.01 U/mL [139]. This sensor was used for a real-sample analysis with a standard deviation of less than 5%. Abou-Omar et al. developed an optical nano-biosensor to detect CA125. The sensor was designed based on nano gold coated by Schiff base doped in a sol–gel matrix, which exhibited fluorescence emission at 430 nm. The fluorescence quenching ability of the CA125 was utilized for the quantification of CA125. A linear response of the sensor in the concentration range of 2–127 U/mL with a LOD of 1.25 U/mL of CA125 was observed. The sensor showed the ability to discriminate between diseased people and the healthy population [140]. Omer et al. exploited the optical properties of carbon quantum dots (CQDs) for the biosensing of CA125. The design of this sensor is based on the quenching efficiency of CD125 relative to the luminescence of CQDs at 535 nm. The sensor performance was monitored within a dynamic range of 0.01–129 U/mL and a detection limit of 0.66 U/mL [141].

### 3.8. IL-10

Interleukin-10 (IL-10) is a cytokine that has been investigated as a potential biomarker for lung cancer. Elevated levels of IL-10 have been observed in patients with lung cancer and it has been found to be associated with poor prognosis [142]. Various methods such as ELISA, RT-qPCR, Luminex assays and some new point-of-care devices have been used to measure IL-10 levels in patient serum, plasma, and tissue samples [143,144]. However, the diagnostic accuracy of IL-10 for lung cancer is limited by its low specificity, as elevated IL-10 levels have also been observed in patients in other cancerous and non-cancerous conditions [145]. Further studies are needed to investigate the diagnostic potential of IL-10 in larger patient populations and to determine its clinical utility for early lung cancer diagnosis.

Tanak et al. developed a novel point-of-care device that simultaneously monitors the immune response by measuring five cytokine biomarkers (IL-6, IL-8, IL-10, TRAIL, and IP-10). The POCT device is composed of a disposable sensor cartridge chip connected to the electrochemical readout setup. The sensor’s sensitivity was significantly enhanced by introducing an array of nanofilm semiconducting/metal electrode interfaces functionalized with specific capture probes to recognize the targets simultaneously by non-faradaic electrochemical impedance spectroscopy. The LOD of the sensor was 1 pg/mL, and analysis of one drop of undiluted plasma took 5 min. Approximately 40 clinical samples were analyzed using this device and its performance was 30 times faster than the standard conventional methods [143]. Stefan et al. reported molecular recognition of IL-8, IL-10, IL-12, and IL-15 by two stochastic sensors developed from modified graphite paste with Ni and Cu complexes of phthalocyanine (PhCN). Interleukins were recognized by their signatures (qualitative parameter) from diagrams obtained after measurements. The LODs of IL-10 from the Ni complex-based sensor and the Cu complex-based sensors were 0.45 ng/mL and 0.45 pg/ mL, respectively. The sensors were validated with various body fluids such as nasal lavage, saliva, serum, and whole blood [144].

Baek et al. used colloidal Au nanoparticles to enhance the localized surface plasmon resonance (LSPR) signal of nano-imprinted Au strips to sensitively detect IL-10. A red shift in the LSPR extinction peak, due to the binding of colloidal Au nanoparticles on the Au strip, enables quantitative detection of IL-10. The sensor was developed using a roll-to-roll nanoimprinting process to generate nano grating on a polyethylene terephthalate (PETp) film. The authors demonstrated a sandwich immune assay using capture and detection antibodies. Colloidal Au nanocubes (AuNC) were crosslinked with IL-10 to enhance the LSPR signal. The sensitivity of the sensor was in nanomolars [146]. Lee et al. developed a novel substrate for quantitative detection of IL-10 using an anti-IL-10 monoclonal antibody. They used 11-(triethoxysilyl)undecanal (TESUD) to functionalize hafnium oxide by chemical vapor deposition and to immobilize antibodies on its surface. The antibody–antigen binding was characterized by a fluorescence pattern, and electrochemical impedance spectroscopy (EIS) was used for further evaluation. A linear response was detected for IL-10 concentrations in the range of 0.1–20 pg/mL and its sensitivity was 0.1 pg/mL [147]. Nessark et al. used substrates composed of polypyrrole (PPy)-modified silicon nitride (Si_3_N_4_) for the label-free detection of IL-10 via a capacitance impedimetric immunosensor in which a human monoclonal antibody—anti-interleukin-10(anti-IL-10 mAb)—was immobilized on the substrate surface. IL-10 was detected via electrochemical impedance spectroscopy (EIS), which showed a linear response range of 1–50 pg/mL and a LOD of 0.347 pg/mL [148]. This simple low-cost electrochemical biosensor can be adopted as a potential choice for the POCT of patients in regions with limited resources.

### 3.9. Vascular Endothelial Growth Factor (VEGF)

VEGF is a 45 kDa homodimeric glycoprotein. It is a potent angiogenic factor that normally promotes angiogenesis during embryonic development in fetuses and during wound healing in adults. Its receptors—VEGF receptor-1 and VEGF receptor-2—are expressed in vascular endothelial cells [149]. The expression of VEGF can be upregulated by a variety of growth factors, hypoxia, and expression of certain oncogenes in cancers. Since tumor development is critically dependent on nutrients and oxygen, the production of VEGF and other growth factors act as an “angiogenic switch,” establishing new vasculature in, and around, the tumor and allowing its rapid growth. However, structural and functional deficiencies in the newly established tumor vasculature can result in progressive hypoxia, which leads to further VEGF production [149].

Biosensors can measure VEGF levels in a short time with low production costs. The results can assist clinicians to predict disease severity in order to address it. Kim et al. developed an impedance biosensor composed of a poly(3,4-ethylenedioxythiophene) (PEDOT)/AuNP composite for detecting VEGF. The nanocomposite was electrochemically deposited into three different configurations: free-standing pads, screen-printed dots, and interdigitated micro-strip electrodes. Anti-VEGF antibodies were immobilized on the polymer films and used to detect VEGF-165 via electrochemical impedance spectroscopy (EIS). A linear relationship was observed between charge transfer resistance (*R_ct_*) and VEGF concentration. The response of the sensor was monitored over a concentration range of 1–20 pg/mL and the LOD of the sensor was found to be 0.5 pg/mL [150]. Sun et al. developed an origami, paper-based, microfluidic, electrochemical device for VEGF biosensing. The working electrode was modified with a nanocomposite made of new methylene blue (NMB), amino-functional single-walled carbon nanotubes (NH_2_-SWCNTs), and AuNPs to enhance its specificity to VEGF. The linear relationship was noted in the dynamic range of 0.01–100 ng/mL with a limit of detection noted at 10 pg/mL. This device was evaluated with clinical serum samples, showing excellent results for real-time detection [151]. Kang et al. developed a fluorescence-linked immunosorbent assay (FLISA) for VEGF biosensing. A low-volume, three-dimensional, microfluidic incubation chamber was used for the complete process of analysis. The process consisted of antigen–antibody binding and fluorogenic substrate binding to the target protein. The sensor detected VEGF at concentrations as low as 1 ng/mL [152]. Kwon et al. demonstrated electrochemical detection of VEGF using a P-type field effect transistor (FET). Polypyrrole nanotubes were conjugated with anti-VEGF RNA aptamers and developed in a cylindrical micelle template in a water–oil emulsion system. The LOD of this FET biosensor was found to be 400 fM [153]. Ghavamipor et al. designed a chemiluminescence enzyme-linked immunosorbent assay (CL-ELISA) for the detection of VEGF using H_2_O_2_-sensitive TGA-CdTe quantum dots as a signal transducer. Dextran and catalase were used to cross-link the antigen and the bioactive reporter enzyme, respectively, allowing for the labeling of the enzyme. This method can detect VEGF in the dynamic range of 2–3500 pg/mL, and the LOD was 0.5 pg/mL, tenfold lower than commercial colorimetric immunoassays. This method was successfully validated using human serum samples and the results were comparable to conventional ELISA [154]. Aptamers are chemical antibodies that are recently being used as sensing agents for the development of biosensors. Nanomaterial-based, optical aptasensors for quantitative detection of VEGF have already been well established using DNA and RNA aptamers. Several aptasensors have been designed based on the principles of electrochemistry, luminescence, fluorescence, colorimetry, and SPR. Their advantages and limitations for practical application have been described elsewhere [155,156]. Wang et al. reported a strip-based fluorescence immunochromatographic (FIC) assay for the detection of VEGF. A quantum dot microsphere-labeled anti-VEGF antibody was used as a fluorescence probe. The fluorescence intensity was proportional to the VEGF concentration in the sample. The detection range of FIC was 25–1600 pg/mL with a LOD of 21 pg/mL [157].

### 3.10. Annexin II

Annexin II is a 38 kDa, calcium-dependent, phospholipid-binding protein. It regulates fibrinolysis, the breakdown of fibrin-containing thrombi, by localizing its proteolytic activity to the cell surface [155]. It does so by serving as a profibrinolytic co-receptor for both plasminogen and tissue plasminogen activator (which is found on the surface of endothelial cells and facilitates the generation of plasmin). Additionally, it plays a key role in regulating other cellular functions, including angiogenesis, proliferation, apoptosis, cell migration, invasion, and adhesion [158]. Recently, Cui and Wang found that annexin II was upregulated in lung cancer tissues and cell lines. It is over-expressed in multiple malignancies and has also emerged as an attractive candidate receptor for plasmin generation on the tumor cell surface [158]. Kim et al. constructed an amperometric immunosensor to detect Annexin II in patient samples. An electrochemical sensor probe was constructed by electropolymerization of conductive polymers (polyterthiophene carboxylic acid, poly TTCA) on the surface of AuNPs/glass carbon electrodes to produce a probe with immobilized dendrimers. Subsequently, anti-Annexin II antibody and hydrazine were covalently linked on the Den/AuNP-modified surface. The sensor exhibited linear dependence in the range of 0.1–1 ng/mL and was able to detect annexin antigens at concentrations as low as 0.05 ng/mL [159]. In another study, Davis et al. developed a radioimmunoassay to detect secreted and intracellular annexin II in human cells. Annexin II is not associated with enzymatic activity, which makes detection difficult. The linear relationship for the sensor was observed up to 0.5 µg/mL, and it was demonstrated that this method can differentiate intracellular and secreted annexin II [160].

### 3.11. ENO1

Enolase-1 (ENO1) is a multifaceted enzyme with oncogenic properties that aids in assessing disease progression. ENO1 not only primarily catalyzes glycolysis, but also mediates intracellular and extracellular processes that vary depending on its location. When localized on the cell surface membrane, it acts as a plasminogen receptor and promotes fibrinolysis and extracellular matrix (ECM) degradation by converting plasminogen into serum serine plasmin. ECM degradation serves as the main driving force for metastasis by stimulating the invasion and migration ability of cells. Additionally, it is also involved in the interphase of the cell cycle by organizing microtubules. ENO1 also regulates gene transcription and translation in cancer cells by displaying multiple binding capacitates to DNA and mRNA [161]. In over 70% of cancer cases, ENO1 was found to be over-expressed, which accelerated the glycolytic pathway and contributed to several tumor progression activities, such as (1) inducing angiogenesis, (2) sustaining proliferative signaling, (3) activating invasion and metastasis, (4) deregulating cellular energetics, and (5) avoiding immune destruction.

Overall, ENO1 is a potent biomarker because of three main qualities. Its surface localization, which makes it accessible upon detection; its significant overexpression in cancer cells; and its positive correlation with worse prognosis and clinical outcomes [162]. It plays a crucial role in activating oncogenic pathways, along with serving as an ideal therapeutic target as cancer cells mainly rely on glycolysis for energy owing to the Warburg effect [162].

Ho et al. reported an electrochemical sandwich immunosensor for the detection of ENO1, in which an anti-ENO1 monoclonal antibody was adsorbed on a polyethylene-glycol-modified disposable screen printer electrode as the capturing probe. The secondary anti-ENO1 polyclonal antibody tagged with AuNPs was used as a detecting probe. The presence of ENO1 resulted in the formation of a sandwich complex that altered the electrochemical signal. The dose response of the sensor was tested in the linear dynamic range of 1 pg/mL to 1 ng/mL with a LOD of 11.9 fg [163]. In another study, Wang et al. performed in vitro, magnetic resonance imaging (MRI) studies using superparamagnetic iron oxide nanoparticles and xenograft models to detect the expression and location of ENO1 in the pancreatic cancer cell lines CFPAC-1 and MiaPaCa-2. ENO1-targeted Dex-g-PCL/SPIO nanoparticles with anti-ENO1 antibodies were constructed. Dex-g-PCL/SPIOns play an important role in the precise detection of pancreatic ductal adenocarcinomas (PDACs) [164]. Alternatively, an electrochemiluminescence immunoassay and amplified luminescent proximity homogeneous assay (AlphaLISA) developed by Yin et al. successfully detected and measured the expression of ENO1 in plasma samples from PDAC patients [165].

### 3.12. Ferritin

Ferritin is an iron-storing protein that is often elevated in lung cancer patients. Elevated serum ferritin in non-small-cell lung cancer (NSCLC) has been attributed to inflammation rather than to body iron overload. Ferritin was also measured in samples from airways such as bronchial secretion and bronchoalveolar lavage (BAL), diagnostic measures for the lower respiratory tract. The source of the ferritin in the airways is suggested to be the transudation of serum iron into airways [166]. In order to quantitively, sensitively, and quickly detect ferritin, Mao et al. developed a cotton thread immunoassay, combined with a novel gold nanoparticle trimer, as a reporter probe to enhance the signal. In this lateral flow immunoassay, a capture anti-ferritin monoclonal antibody was immobilized onto the test zone of the cotton thread. When ferritin and the detection monoclonal antibody were conjugated with AuNps trimers loaded on the conjugate pad, it traveled through the thread, forming a sandwich complex with the capture antibody at the test zone, producing color. The color intensity was proportional to the quantity of ferritin in the sample. The sensor was tested in the ferritin concentration range of 20 ng/mL to 20,000 ng/mL, and it detected as low as 10 ng/mL of ferritin [167]. The same research group has reported a similar, cotton thread-based, immunochromatographic assay for the detection of ferritin using carbon nanotubes as a sensing probe. The sensing range of the method was 100–5000 ng/mL with a LOD of 50 ng/mL [168]. A different research group, Song et al., reported quantitative detection of human ferritin via electrochemical immunoassay by using gold nanorods as sensing probes. This anodic stripping voltametric (ASV)-based device was capable of detecting ferritin in the dynamic range of 5–5000 ng/mL, with up to an LOD of 1.58 ng/mL, with an analysis time of 30 min [169]. In a different study, Wu et al. achieved simultaneous detection of multiple lung cancer biomarkers, including CYFRA21-1, NSE, and ferritin, using electroluminescence on 168 lung cancer patient samples. The results of the CYFRA21-1, NSE, and ferritin analysis revealed that the positive rate of lung cancer was much greater. The worse the clinical stage, the higher their values. Additionally, CYFRA21-1 was higher in squamous carcinoma and NSE was greater in small-cell lung cancer patients. The overall sensitivity of Cyfra21-1, NSE, and ferritin was 91.1% [170]. Alternatively, a novel, highly sensitive, real-time, label-free, horn-like, polycrystalline silicon nanowire field-effect transistor (poly-Si NW FET) immune sensor has also been explored for the detection of ferritin. A poly-Si NW FET using a 2 µM channel length exhibited better performance, such as a small threshold voltage of 1.1 V, compared to other channel lengths. The device showed a LOD of 50 pg/mL of serum ferritin sample in PBS [171].

Garg et al. reported an electrochemical ferritin immunosensor made using biosurfactant-stabilized, and/or functionalized, tungsten disulfide (WS_2_-B) quantum dots (QDs). In this sensor, functionalized WS_2_-B-QDs were used as electroactive probes. Commercially available screen-printed electrodes, functionalized with WS2-B-QD ferritin-specific antibodies, were used for sensor development. Cyclic voltammetry (CV) and differential pulse voltammetry (DPV) were both incorporated into this immunosensor. Ferritin electrochemical sensing in the linear range of 10–1500 ng/mL was accomplished with LODs of 6.048 ng/mL and 3.80 ng/mL for CV and DPV, respectively [172]. This highly sensitive and stable sensor can be applied for POC testing. The use of a micropatterned gold electrode on a silicon chip has also been investigated for ferritin sensing applications. The ferritin and antibody are converted into electrical signals by the micropatterned immunosensor. Rectangular and circular sensors serve as bulk electrodes and microelectrodes, respectively. Both types of electrode calibration curves revealed a linear response of ferritin concentration in the dynamic range of 0.1 g/mL to 1 mg/mL [173]. In one case study, a noninvasive method for the detection of ferritin in exhaled breath condensate (EBC) method was also used. The study included 40 lung cancer patients and 20 chronic obstructive pulmonary disease (COPD) patients, as well as 20 healthy individuals as control. A high level of ferritin was found in lung cancer patients (>60 ng/mL) compared to COPD patients (35–40 ng/mL), and the control group [174]. Alternatively, a paper-based electrochemical immunosensor may be applied to quantitatively detect ferritin. The modification of graphene oxide, on the working electrode, was performed via inkjet printing. Anti-ferritin antibody was immobilized onto the modified electrode’s surface by EDC/NHS chemistry. Ferritin levels were monitored using DPV. The sensor showed a signal response in the ferritin concentration range of 1–1000 ng/mL with a LOD of 0.19 ng/mL. The device was successfully validated with human serum ferritin samples. The device’s stable and repeatable results show that this method could be useful for POC testing in areas with few resources [175]. Garg et al. investigated an alternative, lab-on-a-chip-based electrochemical immunosensor to detect ferritin. By using an electrochemically active SPE, this immunosensor integrates nanotechnology, microfluidics, and electrochemistry. The SPE surface was modified with amine-functionalized graphene oxide to immobilize the anti-ferritin antibody. The setup was then submerged into a microfluidic flow cell, which detects the ferritin continuously. Ferritin detection was monitored via CV in the dynamic range of 7.81–500 ng/mL and the LOD was 0.413 ng/mL. The results from this device were validated with standard ELISA using a spiked human serum sample [176]. An optical biosensor for serum ferritin was developed using using iron oxide nanoparticles (IONP) and photonic crystals (PC) in a biomolecular interaction detection (BIND) system. The system can detect ferritin at concentrations as low as 26 ng/mL. The method was in agreement with the conventional ELISA method [177].

### 3.13. Nitrated Ceruloplasmin

Ceruloplasmin is a 132 kDa glycoprotein that transports copper in the blood. It is synthesized in the liver, from which it transports copper to tissues. Those tissues use copper for metalloenzyme functioning. Its receptors are found on tissues such as the wall of the aorta [178]. Additionally, it can oxidize iron (II) to iron (III), which facilitates the binding of iron to transferrin [179]. Ceruloplasmin is observed to be elevated in lung cancer patients and malignant tumor cells; however, its role in lung adenocarcinoma is still unclear [180]. Li et al. demonstrated a rapid, sensitive, and quantitative detection of nitrated ceruloplasmin using a quantum dot-based lateral flow test strip. When the sample is added, the QD-conjugated detection antibody, in the conjugate pad, complexes with ceruloplasmin nitrate and migrates toward the test line, where it forms a sandwich complex with the immobilized capture antibody. As a result, a fluorescent QDs signal is seen on the test line. The intensity of the test line’s fluorescence is proportional to the concentration of nitrated ceruloplasmin in the sample. This device detected concentrations of nitrated ceruloplasmin as low as 1 ng/mL under optional conditions. Spiked human plasma sample signals were detected across a wide range of concentrations with the LOD of 8 ng/mL. This device allows rapid and sensitive detection of nitrated ceruloplasmin for POC testing [181].

### 3.14. Folate Binding Protein (FBP)

FBP, also known as folate receptor (FR), is a 26.5kDa glycoprotein on epithelial cells. It has a high affinity for folate and mediates folate translocation into cells. Folates are required for the synthesis of nucleotide bases, amino acids, and other methylated compounds. As a result, proliferating cells—such as tumor cells—require them in high quantities. Additionally, FR-α can be used to assess tumor response to anti-folate chemotherapy [182]. FBP detection using a quartz crystal, microbalance biosensor was designed by Henne et al. using a folate-BSA conjugate that was adsorbed onto a gold-coated quartz sensor. The surface was then blocked by a high concentration of folic acid to avoid nonspecific binding to the sensing surface. Gold nanospheres, conjugated with anti-FBP antibody and protein A, increased the sensitivity of the sensor. The LOD of the sensor was improved by three orders of magnitude to 50 pM [183]. A photoelectrochemical (PEC) biosensor based on the antifouling interface and unique ligand–protein recognition might alternatively be used to detect serum-soluble FBP. TiO_2_ nanotube arrays were developed and coated with PDA. The material’s mesoporous nature significantly enhanced the PEC signal. Additionally, antifouling performance was achieved by attaching amino group-terminated 8-chain PEG to the surface. Furthermore, incorporation of FA maintained the sensor’s properties and its FBP recognition ability. The electrochemical signal response of this device was monitored in the concentration range of 0.001–500 ng/mL of FBP and a LOD of 0.0002 ng/mL. The device’s high sensitivity and specificity, with its excellent LOD, make it highly applicable in clinical settings [184].

### 3.15. Alpha-Fetoprotein (AFP)

AFP is a tumor marker used to detect and diagnose certain cancers. Additionally, AFP-initiating pulmonary hepatoid adenocarcinoma is a rare cancer that is not yet been fully studied. To sensitively detect AFP, Gao et al. reported an electrochemical immunosensor that uses a HRP-functionalized AuNR composite (HRPeAuNRs). Secondary (detection) antibodies and HRP were labeled on the surface of the AuNR, whereas the capture antibody was labeled on an arrangement of modified assembling CNTs on a glassy carbon electrode. In the presence of AFP, a sandwich immune complex forms comprised of the capture and detection antibodies by antigen–antibody interaction, causing a clear change in DPV. Under optimal conditions, the signal response of the sensor was reported to be linear over a concentration range of 0.1–100 ng/mL, with a LOD of 30 pg/mL. When the sensor was evaluated with human serum samples, recovery ranged from 90% to 102.7% [185]. In another study, Kim et al. reported a G-FET biosensor for the sensitive detection of AFP in hepatocellular carcinoma (HCC) in humans. The G-FET was functionalized using PBASE and immobilized with an anti-AFP antibody. The sensor detected AFP in both PBS buffer and human plasma at concentrations as low as 0.1 ng/mL and 12.9 ng/mL, respectively, demonstrating potential for clinical application [186]. Shen et al. reported a low-cost MIP electrochemical sensor for AFP cancer biomarkers. AFP-MIP was constructed by coating a glassy carbon electrode with chitosan, GA, and AFP antigen layer-by-layer using surface imprinting in place of an antibody. This sensor exhibited a detection range of 0.8 ng/mL to 10 μg/mL of AFP with a LOD of 96 pg/mL. The sensor performance was evaluated using human serum samples [187].

Xi et al. developed a novel, fluorescent immunosensor that sensitively detects AFP without the use of a fluorophore or enzymes. CuO NPs were labeled with detection antibodies. Capture antibodies then formed sandwich complexes with the detection antibodies in the presence of AFP. The labeled CuO Nps released Cu^2+^, with the help of HCL, which is reduced to Cu^+^ by ascorbate. The Cu^+^ induces a reaction between the weakly fluorescent compound (3-azido-7-hydroxycoumarin) with propargyl alcohol, producing a strongly fluorescent compound. A linear relationship between AFP concentrations (ranging of 0.025–5 ng/mL) and fluorescence intensity was observed, with a LOD of 12 pg/mL. The method was used for human serum sample analysis and yielded reliable results [188]. Phuc et al. developed a MEF method for the detection of AFP cancer biomarkers using gold-capped magnetic (Fe_3_O_4_) nanoparticles (GMPs). The nano-thick gold-coated shell on the core improved the sensor’s fluorescence signal in the linear range of AFP protein concentration from 0.05 to 1000 ng/mL, yielding a LOD of 0.38 pg/mL [189]. Yang et al. developed an immunochromatography test strip (ICTS) and homemade test strips to quickly detect AFP in human serum. The principles of sandwich immunocomplexes were used, with a capture antibody immobilized on a test line and a secondary antibody immobilized on a control line. In the presence of AFP, the quantum-dots-labeled detection antibodies formed a sandwich on the test line, while excess detection antibodies accumulated on the control line. This lateral flow assay could detect AFP at concentrations as low as 1 ng/mL. Compared to the electrochemiluminescence immunoassay AFP kit, the results were in alignment with the standard methods. This rapid, sensitive, and low-cost paper-based strip can be adopted for POC testing for early diagnosis [190]. There are several types of analytical principles that have been used for the detection of AFP using aptamers as recognition receptors, integrated with various transduction methods such as colorimetry, electrochemical luminescence, fluorescence, SPR, photoelectrochemical and SEARS. Many aptasensors have been discussed in recent reviews, which describe the materials used, the method of detection, the detection range, and the LODs [191,192]. Recently, Upan et al. developed an SPGE modified with PtNPs on carboxylated graphene oxide (PtNPs/GO-COOH) as a sensing platform for AFP detection. The AFP-specific aptamer then was immobilized on the sensing platform. The aptamer selectively complexed with AFP and its interaction was investigated using square wave voltammetry. The sensor signal response sensor was monitored over a dynamic range of 3–30 ng/mL and the LOD of the sensor was 1.22 ng/mL. The aptasensor exhibited high selectivity and stability with good recovery of AFP from human serum samples [193].

### 3.16. Serum Amyloid A (SAA)

The acute phase SAA proteins are a collection of 12–14 kDa apolipoproteins found mostly in high-density lipoproteins (HDLs) within the plasma. They are primarily produced by the liver, and, during the acute phase, SAA levels can increase 1000-fold in response to injury, infection, or inflammation [194]. On chromosome 11, four genes encoding for SAA—SAA1, SAA2, SAA3, and SAA4—were discovered [195]. The SAA1 and SAA2 genes encode for the SAA1 and SAA2 proteins, respectively, which together make up the “acute phase” SAA (A-SAA) protein. SAA1 protein accounts for around 70% of A-SAA [196,197]. SAA and C-reactive protein (CRP) are classified as acute-phase proteins because of their presence in acute inflammation at high sensitivity [198]. Lung cancer—now considered to be an over-healed inflammatory condition—has recently been shown to implicate SAA as an acute-phase protein during carcinogenesis. High CRP levels have also been linked to a worse prognosis in patients with lung cancer. CRP-SAA levels were found to be higher in lung cancer patients in one study, with a link between elevation and more severe characteristics, as well as lower overall survival rates [199]. For example, Sung et al. reported SAA1 and SAA2 in the pooled sera of lung cancer patients but not in healthy controls. The expression of SAA1/2 was also greater in lung cancer cells than in normal lung cells. Additionally, incubating lung cancer cells with macrophages boosted the production of IL-1b and IL-6, which further encouraged the lung cancer cells to produce SAA1/2 [200].

Sung et al. created an anodic aluminum oxide (AAO) nanoporous biosensor for rapid and sensitive detection of SAA1 using LSPR coupled with the interferometry technique. The gold-deposited AAO biosensor was made with variable pore sizes ranging from 15–95 nm and a pore depth of 1 µm via a two-step electrochemical anodization process. The pore size was determined by pore-widening treatments based on the anodization condition. The LSPR sensor chip detected changes in the refractive index (RI) of the local environment, providing a method of label-free sensing by immobilizing antibodies on the AAO chip. The complexation of antigen and antibody led to changes in the RI pattern. The LOD of the sensor was 100 ag/mL. This sensor can be applied to monitor real-time interactions of biomolecules [201]. Balayan et al. developed an electrochemical biosensor to detect SAA biomarkers with high sensitivity. It uses a molecularly imprinted polymer method to integrate multiwalled carbon nanotubes (MWCNTs), manganese oxide nanospheres (MNO_2_NSs) and cobalt oxide nanoparticles (Co_3_O_4_NPs) for an efficient synergetic effect and high conductivity over a screen-printed electrode (SPE). The electrode was further modified by the polymerization of molecularly imprinted polymer to sense SAA specifically. The performance of the sensor was tested in an operating range of 0.01 pM to 1 µM and the LOD of the sensor was found to be 0.01 pM [202]. Antibody-coated latex agglutination has also been explored for rapid measurement of human SAA in serum via kinetic nephelometry. SAA-enriched high-density lipoprotein was used as a primary standard for this assay. This assay results correlated well with a conventional enzyme immunoassay. The analysis time of this method was less than 6 min. The operation range of this method was evaluated in the dynamic range of 0.17–10 mg/mL [203].

### 3.17. Neuron-Specific Enolase (NSE)

**Figure 7:**
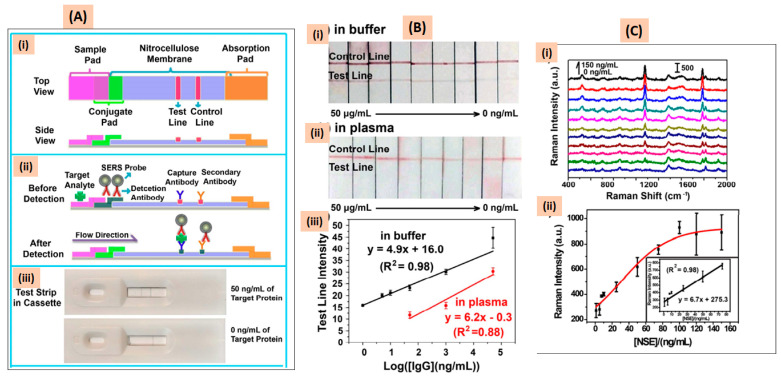
(**A**) Principle and operation mechanism of SERS paper-based lateral flow strip (PLFS). (i) Top view and side view of PLFS; (ii) before and after detection of target protein; (iii) pictures of PLFS cassettes with positive and negative results; (**B**) pictures of colorimetric PLFS and calibration plots for detection of IgG (0~50 µg/mL (i) in PBS buffer and (ii) PBS and blood plasma ratio of 90:10; (iii) calibration curve in PBS (black) and a mixture of PBS and blood plasma (red).; (**C**) (i) SERS spectra (ii) SERS-PLFS with MBA as Raman reporter for detection. Insert: calibration plot obtained from the Raman intensity against NSE concentration. Adapted from [208], with copyright permission under the terms of the CC-BY-NC-ND 3.0 license.

NSE is a potential biomarker for the diagnosis of NSCLC [204]. In SCLC, by contrast, pro-gastrin-releasing peptide (ProGRP) demonstrated the best sensitivity–specificity relationship when compared to NSE and chromogranin A (CGA) [205]. Commercial immunoradiometric assays and ELISA are common techniques used for NSE detection [205]. There are several biosensing methods that can measure NSE levels in body fluids. For example, several studies have been conducted to investigate the use of electrochemical aptasensors for NSE detection in lung cancer patients. Wang et al. reported an electrochemical impedimetric biosensor to detect NSE using a 5,10,15,20-tetra(4-aminophenyl) porphyrin (Zr-TAPP) complex, which has a strong affinity towards Anti-NSE antibodies. Compared to the conventional metal–organic framework, Zr-APP-based biosensors showed outstanding results. This sensor detected NSE in the dynamic range of 100 fg/mL to 2 ng/mL with a LOD of 7.1 fg/mL and also showed very good sensitivity and stability [206]. Toma et al. reported a mussel-inspired, PDA-coated, disposable, silver, plasmonic chip for the rapid and sensitive detection of NSE. The sensor chip was designed by PDA deposition for 20 min to coat its surface, which was made for direct attachment of anti-NSE antibody without conjugating agents. SPF spectroscopy was used to detect NSE using a fluorescence-based sandwich immune assay. The sensor showed a linear relationship with the concentration range of NSE between 1 ng/mL and 100 ng/mL. The LODs of NSE in buffer and diluted human serum were 0.5 ng/mL (11 pM) and 1.4 ng/mL (30 pM), respectively [207]. Gao et al. reported a low-cost, PLFS-based immunoassay for NSE detection using surface-enhanced Raman scattering (SERS) as a signal transducer. The SEAS probe consisted of an integrated Au nanostar, Raman Reporter, and silica sandwich nanoparticles. The SERS-PLFS sensor exhibited both outstanding sensitivity and LOD compared to conventional colorimetric PLFS assay. NSE detection from the diluted blood samples was demonstrated with the LOD of 0.08 ng/mL. The sensor performance was monitored in the dynamic range of 1 ng/mL to 0.05 mg/mL. This method can be useful in point-of-care applications Figure 7 [208]. Zhang et al. reported a label-free electrochemical immunoassay for the detection of NSE using a three-dimensional microporous, reduced graphene oxide/polyaniline (3DM rGO/PANI) film. This 3DM rGO/PANI was prepared by the co-electrodeposition of GO and aniline. The GO was reduced to rGO and PANI was then deposited on the surface of rGO sheets. The ratio of rGO and PANI was optimized to improve the performance of the sensor. The 3DM rGO/PANI had a high surface area that could facilitate the immobilization of antibodies, high conductivity, and electron transfer. Under optimal conditions, the sensor response was monitored in the linear range of 0.5 pg/mL to 10 ng/mL with a measured LOD of 0.1 pg/mL. As it is highly specific and sensitive, this setup can be applied clinically [209]. Li et al. have also developed a disposable, point-of-care, electrochemical immunosensor that allows for rapid detection of NSE. Fe_3_O_4_ and CuS nanoparticles were used as substrates to capture Ab1 and the labeled reporter antibody, Ab2, respectively. This method did not need a washing step, and a syringe filter was used for sample preparation. CuS Ab2 passed through 200 nm pores of the filter while the larger immunocomplex—Fe_3_O_4_-Ab1/NSE/CuS-Ab2—was blocked. CuS-Ab2 generated a current via electron transfer between Cu^2+^ and Cu^+^ at the gold electrode. The immunosensor’s response was evaluated for NSE concentrations ranging from 100 fg/mL to 50 ng/mL, while the observed LOD of the sensor was 33 fg/mL, which would allow its application [210].

**Figure 8:**
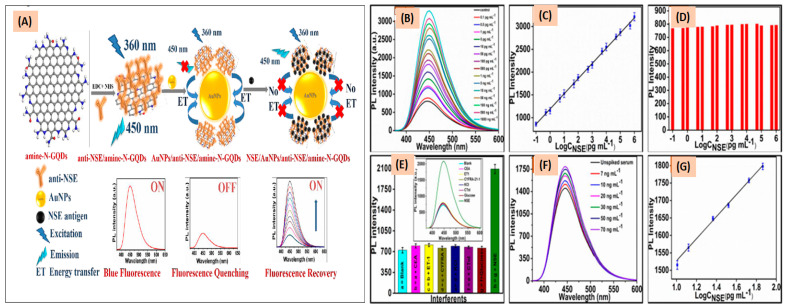
(**A**) Illustration of the fluorescent biosensor working principle for small-cell lung cancer biomarker detection; (**B**) dose-dependent fluorescence response of anti-NSE/amine-N-GQDs@AuNps nanoprobe as a function of NSE concentration in the range of 0.1 pg mL^−1^ to 1000 ng/mL from bottom to top); (**C**) the calibration plot obtained by plotting log concentration of NSE and PL intensity of the nanoprobe indicating the linear relationship; (**D**) control experiment of amine-N-GQDs@AuNps performed without anti-NSE antibody; (**E**) cross-reactivity test of anti-NSE/amine-N-GQDs@AuNps nanoprobe with other potential interfering biomolecules; (**F**) change in the fluorescence response of anti-NSE/amine-N-GQDs@AuNps nanoprobe with the NSE spiked serum samples (7, 10, 20, 30, 50, and 70 ng/mL); (**G**) the calibration plot of spiked serum samples shows the linear relationship of log concentration of NSE against PL intensity of the nanoprobe. Adapted from [214], with copyright permission.

Aptasensors are highly sensitive and specific, making them promising tools for SCLC diagnosis and monitoring. However, because the majority of studies were conducted in vitro, or ex vivo, on patient and spiked samples, extra validation is required [211]. Shen et al. developed an aptamer-based surface plasmon resonance (SPR) assay for the direct detection of NSE. First, the aptamers were immobilized onto the SPR sensor chips. When the sample was introduced, a change in the SPR signal was observed due to NSE-aptamer complex formation. The changes in the signal were observed to be directly proportional to the NSE concentrations and were observed in the range of 3.9 nM to 1 µM; the sensor detected NSE at concentrations as low as 3.9 nM [105]. Zheng et al. also selected NSE-specific aptamers and applied them for the development of a chemiluminescent aptasensor to detect NSE in serum. First, aptamers were immobilized onto magnetic beads and then incubated with NSE. Then, the primary mouse NSE antibody and alkaline phosphatase (ALP)-labeled secondary goat antimouse antibodies were also incubated. The chemiluminescence of AMPPD triggered by complex formation was detected. The sensor was tested with both standard NSE as well as serum samples, and the aptasensor was tested in the dynamic range of 1–100 ng/mL with an observed LOD of 0.1 ng/mL [212]. A label-free FET-based biosensor for the simultaneous detection of NSE and CYFRA 21-1 has been constructed where NSE and CYFRA21-1 antibodies were immobilized onto the same sensor chip. This sensor was able to detect both NSE and CYFRA21-1 in a wide range of concentrations with LODs of 1 ng/mL and 10 ng/mL for CYFRA21-1 and NSE, respectively [213]. In another study, Kalkal et al. developed an ultrasensitive detection of small-cell lung cancer biomarker, NSE, from the FRET method, in which the bifunctional graphene quantum dots act as energy donors and AuNps act as acceptors. They synthesized amine-functionalized and nitrogen-doped graphene quantum dots (amine-N-GQDs) for the sensor construction. Anti-NSE monoclonal antibody was immobilized on amine-N-GQDs to obtain biofunctionalized QDs, anti-NSE/amine-N-GQDs for sensitive biosensors based on the nanosurface energy transfer (NSET) from anti-NSE/amine-N-GQDs to AuNps for NSE detection. The dose-dependent fluorescence responses of anti-NSE/amine-N-GQDs@AuNPs nanoprobes as a function of NSE were measured in the of range of 0.1 pg mL^−1^ to 1000 ng/mL with a LOD of 0.09 pg/mL. The real sample analysis showed an outstanding performance with an average recovery of 94.69%. The design and construction of GQDs and the results of the quantitative detection of NSE are illustrated in Figure 8 [214].

### 3.18. Squamous Cell Carcinoma Antigen (SCCA)

SCCA is a protein that is expressed by normal squamous cells. It is often used as a biomarker for squamous cell carcinomas, as it is known to be elevated in various types of squamous cell carcinomas, including those affecting the lung, cervix uteri, head and neck regions, and esophagus. In lung cancer specifically, SCCA levels have been found to be closely related to the stage of the cancer, with higher levels indicating more advanced disease [215]. Recently, Wu et al. reported a novel “in-electrode” type of ECL biosensor to detect SCCA. The sensing matrix consisted of magnetic graphene oxide (Fe_3_O_4_@GO), which effectively captured Ab1, greatly amplifying the signal. AuNPs/g-C_3_N_4_ was also used as the signal tag, which not only improved the loading capacity of Ab2, but also enhanced conductivity for improved ECL intensity. The ECL biosensor demonstrated a low LOD of 0.4 pg/mL [216]. Similarly, Mo et al. developed an ECL method for the sensitive detection of SCCA using combined molecular imprinting by electropolymerization and Fe(III)-MIL-88B-NH_2_ MOFs to load ZnSeQDs. In this construction, uniform PDA films were first prepared by electropolymerization, and ZnSeQDs were encapsulated in Fe(III)-MIL-88B-NH_2_, creating Fe(III)-MIL-88B-NH_2_@ZnSeQDs. Subsequently, SCCA antibody was immobilized onto the surface to create an antibody-capturing signal probe. Due to their large surface area, MOFs were used as a carrier for the high loading of ZnSeQDs. Fe(III)-MIL-88B-NH_2_@ZnSeQDs/Ab acted as a co-reaction accelerator to facilitate the conversion of S_2_O_8_^2−^ to SO^4−^, producing a high-intensity electrochemical luminescence. The sensor performance was tested with SCAA in the linear range of 0.0001-100 ng/mL with a LOD of 31 fg/mL [217]. Zhao et al. also designed a novel, reusable LSPR method for the sensitive detection of SCCA. In this sensor, a triangle-shaped silver nanoparticle array was developed using nanosphere lithography, followed by the formation of a self-assembled monolayer (SAM), on the surface by 11-mercaptoundecanoic acid (MUA). Monoclonal anti-SCCA antibodies were immobilized onto the SAM layer of the sensor chip by EDC/NHS chemistry for the detection of SCC antigen. The variable concentration of SCCA was tested both in buffer and in human serum in the linear range from 0.1 to 1000 pM. The LOD was 0.125 pM. The used sensor was regenerated by using a suitable regeneration solution (Ex: 50 mM glycine-HCl (pH 2.0)) [218].

### 3.19. Heat Shock Proteins

Heat shock proteins (HSPs) are a class of highly conserved molecular chaperons. HSPs are known to be expressed ubiquitously in prokaryotic and eukaryotic cells and play an important role in protein folding, protein conformational stability, and cellular homeostasis. HSPs are classified based on their molecular weight into different classes including HSP100, HSP90, HSP70, HSP60, and small HSPs such as Hsp33 and Hsp27 [219].

In several malignancies, including lung cancer, HSPs are overexpressed, and this enhanced expression plays a crucial role in protecting tumor cells from spontaneous apoptosis associated with malignancy as well as the apoptosis generated by chemotherapy [220]. The essential role of HSPs and how they interact with intracellular signaling in lung cancer cells were discussed in depth by Mittal and Rajala [221]. HSPs have been found to collaborate with different oncogenes, including tyrosine kinases (v-Src, Bcr/Abl), and promote cancer growth [222,223]. In addition, there is inhibition in apoptotic pathways and senescence, while cell survival is promoted in cells that express high levels of HSPs [221,224]. These findings suggest that cancer cells might require the expression of HSPs for their growth and survival. Wang et al. demonstrated that HSP90α expression in the serum of patients with non-small-cell lung carcinoma was higher than in small-cell lung carcinoma. Moreover, HSP90α expression was higher in lung cancer patients’ serum in comparison to healthy individuals’ serum, indicating that HSP90 could be employed as a novel biomarker for the diagnosis of lung cancer [225]. A recent study by Fang et al. found that HSP90α expressions were positively correlated with tumor–node–metastasis staging in patients with lung adenocarcinoma and suggested combining HSP90α with carcinoembryonic antigen for more effective prediction of patient prognosis [226]. The growing evidence that shows a link between HSP expression and lung cancer differentiation and staging suggests that HSPs can be used as a potential biomarker and therapeutic target to treat lung cancer.

Two strategies are under consideration to target HSPs since they play a regulatory function in both physiological and pathological settings. The first is to modulate their expression level and activity, while the second is to develop HSP-based immunotherapies. No pharmacological drug has yet been clinically approved to control the molecular expression and activity of HSPs. However, several HSPs inhibitors completed their clinical trials in the setting of lung cancer.

The HSP90 inhibitor (AUY922) was examined in a phase 2 trial in advanced non-small-cell lung cancer [227]. The efficacy results show that almost 76% of the enrolled patients had no clinical benefit, only 13% achieved investigator-assessed tumor response, and 11% had a stable state. The phase I clinical trial of the HSP90 inhibitor (SNX-5422) assessed the safety and efficacy of using SNX-5422 along with carboplatin and paclitaxel in patients with advanced lung cancer [228]. They reported that it was well-tolerated and showed anti-tumor activity. However, further studies are needed to assess the safety and efficacy of HSP90 inhibitors in lung cancer settings.

On the other hand, early-stage detection of HSP70 cancer biomarkers is important for timely treatment and prevention of further tumor growth. In this regard, Maniya et al. designed and fabricated a form of label electrochemical detection of HSP70 on a low-cost plastic chip electrode (PCE) platform. The gold-coated PCE was modified with 4-aminothiophenol and glutaraldehyde conjugation. On top of this layer, anti-HSP70 was immobilized. Differential pulse voltammetry (DPV) was used for the detection of the target molecule. The change in the current in the presence of HSP70 was utilized for the quantification. The sensor was performed in the concentration range of 0.01–1000 ng/mL with a LOD of 3.5 pg/mL. The performance of the sensor was demonstrated with HSP70 containing serum samples [229]. Pohanka used a quartz crystal microbalance (QCM) biosensor as a sensing tool for the detection of HSP60. The principle of QCM assay was based on the sandwich formation with the antibodies in the presence of the HSP60 target. QCM coated with HSP60 specific capture antibody and modified AuNp as the detection antibody were used. The assay was performed with a low volume of the sample, 5 µL in the HSP60 concentration range of 10 pg/mL to 100 µg/mL with a LOD of 83 pg/mL and an analysis time of less than 90 min. The results from this sensor were in good agreement with the standard ELISA [230]. An indium tin oxide (ITO) based disposable immunosensor has been developed for the early-stage detection of HSP70. In this assay, AuNp modified ITO coated polyethylene terephthalate (PET) electrodes were used for sensor construction. In the presence of cysteamine, a self-assembled monolayer forms on the AuNps that was used for the immobilization of the antiHSP70 antibody-modified ITO surface. [Fe(CN)_6_]^4−^ and [Fe(CN)_6_]^3−^ were used as the redox probe. The electrochemical signal change in the presence of HSP70 was monitored from 1 to 166 fg/mL, and the sensor can detect HSP70 at concentrations as low as 0.0618 fg/mL [231]. These low-cost disposable HSP biosensors would be an alternate tool for point-of-care applications to monitor lung cancer patients in remote and limited-resource settings.

Different kinds of biosensors used for the detection of various biomarkers, materials used, recognition elements, working ranges of sensors, limits of detection, and the respective references are summarized in Table 1.

## 4. Conclusions and Future Prospects

Identification of novel diagnostic, prognostic, and predictive biomarkers is very important to improve the survival rate of lung cancer patients. Liquid biopsy is an ideal method for cancer diagnosis that includes heterogeneity and is non-invasive, making it superior to single-tissue biopsies. Early-stage diagnosis of lung cancer biomarkers is crucial for a clear understanding of pulmonary malignancies and to administer appropriate treatments. The main challenge for biomarker diagnosis is their low abundance in body fluids; in some cases, the biomarker abundance is less than 1 ng/mL. Therefore, the biosensors must be highly sensitive and specific to their targets. Different methods have been developed for early diagnosis of lung cancer, to assess prognosis, and for timely treatment. However, they show certain disadvantages, such as invasive sample collection, long analysis time, high cost, and susceptibility to the age and physical condition of the patient. Biosensor technology holds great promise to become a more reliable alternative tool for the early diagnosis of lung cancer and to reduce mortality rates. Several advanced, highly sensitive and specific biosensors have been reported that allow non-invasive, portable, and cost-effective screening and diagnosis. Lung-cancer-specific nucleic acid biomarkers, including methylated DNA and miRs, can be detected using different nucleic acid amplification techniques such as PCR, RCA, EXPAR, etc. These methods require a complex primer design, highly sophisticated equipment, and well-trained professionals. LAMP is a simple nucleic acid amplification technique that does not need a laboratory, and the results can be visualized by a change in the color of the sample. In addition, simple optical biosensors that can detect miR and methylated DNA with high sensitivity have been developed, enabling point-of-care testing. For example, Feng et al. developed a miR amplification method that can predict the level of miR with a simple pH test paper [82]. Though the end results can be predicted from the pH test paper, the sample analysis was still through the netlike rolling circle amplification (NRCA) technique that needs a minimal laboratory setting. Compared to nucleic-acid-based biomarker diagnosis, protein-based biomarker detection and serology tests are convenient for rapid, cost-effective, and less laborious tests. Despite many advantages including ASSURED and POC testing, low sensitivity and cross-reactivity interference remain major limitations in protein-based diagnosis. There are also many biosensors developed for the sensitive detection of biomarkers with very low detection limits and low sub-picomolar/femtomolar ranges. Regardless of high sensitivity and specificity, designing the sensors in a portable form for point-of-care applications is still challenging. As such, electrochemical biosensors offer more sensitive, specific, and low detection limits while using biological fluids obtained from non-invasive processes. They are relatively easy to create and remain stable for longer periods. Nanomaterials with high conductivity and a greater surface area to volume ratio can be used to enhance the electrochemical signal, which might help detect trace amounts of cancer biomarkers in body fluids. However, false positive and false negative results that occur due to the non-specific adsorption of biomolecules remain a big challenge. In addition, further improvements have to be made to integrate nanomaterials for the making of miniaturized electrochemical sensors for point-of-care testing [232]. Paper-based, semi-quantitative, colorimetric assays for biomarker detection would be ideal to assess the severity and the stage of the cancer, allowing for self-monitoring outside of the clinic, and can help patients make decisions about taking specific treatments. Information from a single biomarker is still not sufficient for a physician to determine the cancer stage, appropriate treatment, and other details of patient’s condition. Therefore, multiple biomarker detection from the same sample is required, which can be achieved using biosensors with multiplex designs that can detect multiple biomarkers. This can reduce the rate of false positives and false negatives [73,233]. The limitations of biosensors in real sample analysis includes small target size, interference from non-specific molecules, and commercial availability. Nanomaterial-based biosensors for the detection of cancer biomarkers have great potential in the future [234]. Although many nanomaterial-based sensors have been reported for lung cancer biomarkers, there are still many changes to be addressed. As of yet, these methods remain too immature for clinical trials and more research has to be performed to improve their sensitivity, accuracy, and the multiplexing capacity of biosensors for clinical applications.

## Figures and Tables

**Table 1:** The biosensor technologies designed with various biomaterials for the sensitive detection of lung cancer biomarkers.

Analyte	Transduction Method	Materials Used	Recognition Element	Linear Range	LOD	Reference
Adenosine	Electrochemical aptasensors	Indium tin oxide (ITO) coated glass electrode modified with (3-aminopropyl)triethoxysilane (APTES) and 3-thiophenecarboxylic acid (3-Th-COOH) followed by electrochemical polymerization of EDOT and ProDOT-(COOH)_2_	Aptamer	9.6 nM to 600 µM	2.33 nM	[91]
ATP	Electrochemical aptasensors	Graphene and gold nanoparticle (AuNP) modified carbon paste electrode (Gr–AuNP–CPE)	Aptamer	0.114 nM–30 µM	20 pM	[93]
ATP	Surface plasmon resonance (SPR) aptasensors	Cytosines (C) modified aptamer in the stem part of the stem–loop aptamer	Aptamer	0.05–500 nM	15 pM	[97]
Adenosine	Luminescence resonance energy transfer (LRET) aptasensors	Tb^3+^ chelated complex labeled DNA, quencher labeled DNA, and extended aptamer DNA	Aptamer	-	60 µM	[99]
Adenosine	Carbon dot nanoparticle induced chemiluminescence aptasensors	Aptamer-functionalized graphene@magnetic β-cyclodextrin polymers	Aptamer	0.5 pM–5 nM	0.21 pM	[100]
Adenosine	Electrochemical aptasensor	Co-assembling of thiolated aptamer, dithothreito (DTT) l, and 6-mercaptohexanol (MCH) on the surface of the gold electrode (Au/aptamer-DTT/MCH)	Aptamer	0.05 pM to 17 pM	0.02 pM	[94]
ProGRP_31–98_	Electrochemiluminescence	Aptamer-modified electrode	Aptamer	0.48–3.36 µM	17 nM	[104]
ProGRP_31–98_	Surface plasmon resonance (SPR) aptasensors	Aptamer-modified SPR sensor surface	Aptamer	15.6 nM–1 µM	15.6 nM	[105]
Pro-GRP	Photoelectrochemical sensor	Electrochemically deposited molecularly imprinted polymerized ionic liquid film on a AuNP/2D-MoS_2_/GCE surface	PMIMBr ionic liquid functional monomer/Pro-GRP template.	0.02–5 ng/mL	0.0032 ng/mL	[106]
Pro-GRP	Electrochemical immunosensor	Modified electrodes using 3D- rGO gold nanoparticles composite (3D-rGO@Au) substrate and SiO_2_ nanosphere-modified detection antibody	Antibodies	1 fg/mL to 10 ng/mL	0.14 fg/mL	[107]
Pro-GRP	Electrochemicalimmunosensor	Secondary antibody labeled with ferrocene and GOD) linked to nano-Au/TiO_2_ capture antibody immobilized on Cys/GS-Nf membrane	Antibodies	10–500 pg/mL	3 pg/mL	[108]
CYFRA21-1	Electrochemical sensors	3,4,9,10- perylenetetracarboxylic dianhydride (PTCDA)@C60 and gold nanoparticles (Au NPs) modified indium tin oxide (ITO) electrode	Antibodies	0.001–40 ng/ml	0.3 pg/mL	[112]
CYFRA21-1	Photoelectrochemical sensor	3,4,9,10- perylenetetracarboxylic dianhydride (PTCDA)@C60 and gold nanoparticles (Au NPs) modified indium tin oxide (ITO) electrode	Antibodies	0.0001–4 ng/mL	0.03 pg/mL	[112]
CYFRA21-1	Electrochemical immunosensors	Bovine serum albumin/anti-CYFRA-21-1/(3-aminopropyl) triethoxysilane/TiO_2_/indium tin oxide immunoelectrode	Antibody	0–12 ng/mL	0.24 ng/mL	[113]
CYFRA21-1	Surface plasmon resonance (SPR)	Carboxyl-functionalized molybdenum disulfide (carboxyl-MoS_2_) nanocomposites-modified surface plasmon resonance (SPR) sensing surface	Antibody	0.05 pg/mL–100 ng/mL	0.05 pg/mL	[114]
CYFRA21-1 DNA	Electrochemical biosensor	Carboxyl-functionalized graphene oxide (GO-COOH) and copper oxide nanowires (CuO NWs) nanocomposite	cDNA probe	1 µM to 1 pM	0.118 pM	[116]
CYFRA21-1	Electronic biosensor	Reduced graphene oxide (rGO)/melamine (MEL)/antibodies/bovine serum albumin (BSA)	Antibody	1 pg/mL to 800 ng/mL	0.04 pg/mL	[117]
CYFRA21-1	Electrochemiluminescence immunosensor	Electrochemically induced atom transfer radical polymerization (eATRP)	Antibody	1 fg/mL to 1 μg/mL	0.8 fg/mL	[118]
CYFRA21-1	Immunofluorescence sensor	Carbon dots decorated with ZnO nanocomposite as a fluorescence probe	Antibody	0.01–100 ng/mL	0.008 ng/mL	[119]
CYFRA21-1	Immunochromatographic sensor	Fluorescent europium chelate nanosphere-based immunochromatographic test strip	Antibody	0–100 ng/mL	1.164 ng/mL	[120]
CEA	Photoelectrochemical immunosensor	Pillar [5] arene functionalized Au and polyaniline–bismuth oxybromide heterojunction (Au@WP5/PANI-BiOBr)	Antibody	0.01–50 ng/mL	3 pg/mL	[126]
CEA	Electrochemical immunosensors	Metal–organic framework (Ce-MOF) skeleton precursor coated with hyaluronic acid (HA). Silver nanoparticles (Ag NPs) and horseradish peroxidase (HRP)-modified antibody. (Ce-MoF @HA/Ag-HRP-Ab2)	Antibody	1 pg/mL to 80 ng/mL	0.02 pg/mL	[127]
CEA	Electrochemical immunosensors	Magnetic-force-assisted electrochemical sandwich immunoassay (MESIA)	Antibody	0.5–200 ng/mL	0.5 ng/mL	[128]
CEA	Electronic biosensor	Reduced graphene oxide (rGO)/melamine (MEL)/antibodies/bovine serum albumin (BSA)	Antibody	1 pg/mL to 800 ng/mL	0.148 pg/mL	[117]
CEA	Flexible free-standing electrochemical biosensor	AuNps were deposited on the polypyrrole (PPy) nanocomposite film electrode	Aptamer	0.1 ng/mL to 1 µg/mL	0.033 ng/mL	[129]
CEA	Fluorescence aptasensor	CuNps intercalated into the dsDNA duplex between CEA aptamer and cDNA aptamer	Aptamer	0.01–2 ng/mL	0.0065 ng/mL	[131]
CEA	Fluorescence sensor	DNA walker cascade amplification strategy of exonuclease (Exo) III-assisted target recycling amplification (ERA). N-methylmesoporphyrin IX (NMM) assisted fluorescence signals		10 pg/mL to 100 ng/mL	0.12 pg/mL	[132]
CEA	Paper-based colorimetric lateral flow assay	Polydopamine-modified AuNps	Antibody	0.05 to 50 ng/mL	0.05 ng/mL	[133]
CEA	SPR immunosensors	Biofunctionalized AuNPs	Antibody		0.1 ng/mL	[134]
CA-15-3	Electrochemical immunosensor	mercaptosuccinic acid self-assembled on gold screen-printed electrode (AuSPE) surface	Antibody	1–1000 U/mL	0.95 U/mL	[138]
CA19-9	Electrochemical immunosensor	Hybrid self-assembled membrane modified with a gold electrode	Antibody	0.05–500 U/mL	0.01 U/mL	[139]
CA-125	Fluorescence sensor	Nano-gold coated by Schiff base doped in a sol–gel matrix	Quenching efficiency of CD125	2 to 127 U/mL	1.25 U/mL	[140]
CA-125	Fluorescence resonance energy transfer (FRET)	Carbon quantum dots (CQDs), fluorescence quenching by CA125	Quenching efficiency of CD125	0.01 to 129 U/mL	0.66 U/mL	[141]
IL-10	Electrochemical biosensor	An array of nanofilm semiconducting/metal electrode interfaces, functionalized with specific capture probes	Host immune response		1 pg/mL	[143]
IL-10	Phthalocyanine-based stochastic sensors	Modified graphite paste with Ni and Cu complexes of phthalocyanine (PhCN)	Ni and Cu (PhCN) complexes	4.5 × 10^−7^–4.5 × 10^−15^ g/mL	45 ng/mL for Ni 45 pg/mL for Cu	[144]
IL-10	Localized surface plasmon resonance (LSPR)	Colloidal gold (Au) nanoparticle/nanoimprinted Au strips (roll-to-roll nanoimprinting process to generate nano grating on the polyethylene terephthalate (PET) film)	Antibodies	-	-	[146]
Il-10	Electrochemical biosensor	Hafnium oxide was functionalized using 11-(triethoxysilyl) undecanal (TESUD) by chemical vapor deposition	Antibody	0.1–20 pg/mL	0.1 pg/mL	[147]
Il-10	Electrochemical biosensor	Polypyrrole (PPy)-modified silicon nitride (Si_3_N_4_) substrates	Antibody	1 to 50 pg/ml	0.347 pg/mL	[148]
VEGF	Electrochemical immunosensor	Poly(3,4-ethylenedioxythiophene) (PEDOT)/gold nanoparticle (Au NP) composite	Antibody	1–20 pg/mL	0.5 pg/mL	[150]
VEGF	Origami-paper-based electrochemical immunosensor	New methylene blue (NMB), amino-functional single-walled carbon nanotubes (NH_2_-SWCNTs), and gold nanoparticles (AuNPs) nanocomposite	Antibody	0.01 ng/mL and 100 ng/mL	10 pg/mL	[151]
VEGF	Field-effect transistor	Polypyrrole nanotubes conjugated with anti-VEGF RNA aptamer	Aptamer		400 fM	[153]
VEGF	Fluorescence-linked immunosorbent assay (FLISA)	Three-dimensional microfluidic incubation chamber	Antibody		1 ng/mL	[152]
VEGF	(CL-ELISA)	H_2_O_2_-sensitive TGA-CdTe quantum dots	Antibody	2–3500 pg/mL	0.5 pg/mL	[154]
VEGF	Fluorescence immunochromatographic	Quantum-dot-microsphere-labeled anti-VEGF antibody was used as a fluorescence probe	Antibody	25 to 1600 pg/mL	21 pg/mL	[157]
Annexin II	Electrochemical immunosensor	Electropolymerized conducting polymer (poly-terthiophene carboxylic acid, poly-TTCA) on AuNPs/glassy carbon electrode/dendrimer/anti-Annexin II antibody and hydrazine on the Den/AuNP-modified surface	Antibody	0.1–1 ng/mL	0.05 ng/mL	[159]
ENO1	Electrochemical sandwich immunosensor	Polyethylene-glycol-modified disposable screen printer electrode	Antibody	1 pg/mL to 1 ng/mL	11.9 fg/mL	[163]
Ferritin	Lateral flow immunoassay	Anti-ferritin monoclonal antibody immobilized cotton thread/gold nanoparticle trimer–antibody conjugate	Antibody	20 to 20,000 ng/mL	10 ng/mL	[167]
Ferritin	Immunochromatographic assay	Anti-ferritin monoclonal antibody immobilized cotton thread/MWCNT-antibody conjugate	Antibody	100–5000 ng/mL	50 ng/mL	[168]
Ferritin	Electrochemical immunoassay	Anti-ferritin monoclonal antibody immobilized cotton thread/gold nanorod and antibody conjugates (GNR-dAb)	Antibody	5–5000 ng/mL	1.58 ng/mL	[169]
Ferritin	Field-effect transistor	Horn-like polycrystalline silicon nanowire field-effect transistor (poly-Si NW FET)	Antibody	50 pg/mL–500 ng/mL	50 pg/mL	[171]
Ferritin	Electrochemical immunosensor	Biosurfactant stabilized/functionalized tungsten disulfide (WS_2_-B) quantum dots (QDs). CV and DPV methods are used	Antibody	10–1500 ng/mL	6.048 ng/mL (CV) 3.8 ng/mL (DPV)	[172]
Ferritin	Electrochemical immunosensor	Immunoprecipitation and electrochemical signaling principle in microfabricated electrodes	Antibody	0.1 ug/mL–1 mg/mL		[173]
Ferritin	Paper-based electrochemical immunosensor	GO modified working electrode by inkjet printing	Antibody	of 1 to 1000 ng/mL	0.19 ng/mL	[175]
Ferritin	Lab-on-a-chip/electrochemical immunosensor	Amine-functionalized GO-modified screen printing electrode (SPE)/submerged in microfluidic flow cell	Antibody	7.81 to 500 ng/mL	0.413 ng/mL	[176]
Nitrated ceruloplasmin	Lateral flow test strip fluorescence assay	Quantum-dot-conjugated antibody as a fluorescent probe	Antibody	1 ng/mL–10 µg/mL	1 ng/mL 8 ng/ml	[181]
FBP	Quartz crystal microbalance biosensor	Folate-BSA conjugate adsorbed on the gold-coated quartz sensor			30 nM	[183]
FBP	Photoelectrochemical	Polydopamine (PDA) coated TiO_2_ nanotube array/amino-group-terminated 8-arm poly(ethylene glycol) (PEG)-Folic acid	Folic acid	0.001 to 500 ng/mL	0.0002 ng/mL	[184]
AFP	Electrochemical immunosensor	Carbon nanotubes (CNTs) modified glassy carbon electrode/HRP functionalized gold nanorods (AuNR) composite (HRPeAuNRs)	Antibody	0.1 to 100 ng/mL	30 pg/mL	[185]
AFP	Graphene field-effect transistor (G-FET)	1-pyrenebutyric acid N-hydroxysuccinimide ester (PBASE) functionalized G-FET	Antibody		0.1 ng/mL	[186]
		chitosan, glutaraldehyde (GA) and AFP antigen layer-by-layer coating on the glassy carbon electrode by the surface imprinting procedure in the place of the antibody (AFP-MIP)	AFP-MIP	0.8 ng/mL to 10 μg/mL	96 pg/mL	[187]
AFP	Fluorescent immunosensor	CuNp-induced reaction between a weak fluorescent compound (3-azido-7-hydroxycoumarin) and propargyl alcohol produces a strongly fluorescent compound	Antibody	0.025 to 5 ng/mL	12 pg/mL	[188]
AFP	Metal-enhanced fluorescence sensor	Gold-capped magnetic (Fe_3_O_4_) nanoparticles (GMPs)/enhanced fluorescence signal	Gold-capped magnetic (Fe_3_O_4_) nanoparticles (GMPs)	0.05 to 1000 ng/mL	0.38 pg/mL	[189]
AFP	Electrochemical aptasensor	Screen-printed graphene–carbon paste electrode (SPGE) modified with Platinum nanoparticles on carboxylated-graphene oxide (PtNPs/GO-COOH)	Antibody	3 to 30 ng/mL	1.22 ng/mL	[193]
Methylated DNA	Electrochemical biosensor	Stem–loop structured DNA probe labeled with thiol and methylene blue	cDNA probe	-	4 fM	[42]
p53 gene methylation	Electrochemical sensor	Peptide nucleic acid (PNA)	cPeptide nucleic acid (PNA)	50 pM–96 nM	18 pM	[44]
Gene-specific DNA methylation	Electrochemical sensor	Fe_3_O_4_/Ntrimethyl chitosan/gold (Fe_3_O_4_/TMC/Au) nanocomposite for tagging the DNA probe and polythiophene as sensing element	cDNA probe	10 fM–5 nM	2 fM	[45]
Site Specific DNA methylation	Electrochemical sensor	Anti-5-methylcytosine antibody was immobilized with GO/horseradish peroxidase-labeled IgG (HRP-IgG)	Antibody	1 fM–10 nM	1 fM	[49]
DNA methylation	Colorimetry	NaCl-induced AuNp aggregation	Complementary DNA probe	80 fM to 80 pM	80 fM	[51]
Cytosine methylation sites	FRET	FRET between upconversion nanoparticles (UCNP) and gold nanorods (AuNR)	Complementary DNA probe	-	7 pM	[59]
MTase activity	Fluorescence sensor	SYBR Green-1/dsDNA interaction in the presence of carbon nanoparticles	Intercalation of SYBR Green1in dsDNA duplex	0.5–100 u/mL	0.1 u/mL	[61]
methylated DNA	Fluorescence sensor	Capped CdTe quantum dots as a fluorescence prob	quantum dot intercalation into unmethylated DNA	0.1 nM–1 µM	60 pM	[62]
Mir10b Mir16 Mir191	FRET sensors	Competitive DNA displacement: DNA labeled with FAM-labeled cDNA/D/short DNA labeled fluorescence quencher	cDNA/MiR hybridization	0.2–250 nM	MiR10b: 97 pM, MiR16; 10, Mir 191: 1 pM	[70]
MiR21 and miR205	Personal glucometer	Duplex-specific nuclease-assisted CRISPR-Cas12a/sucrose to glucose/Glucometer	Complementary DNA	10 pM–100 nM	MiR21; 2.4 pM Mir205: 1.1 nM	[71]
MiR21, MiR 141 and Let-7a	Digital flow cytometry	Digital flow cytometry-ligation rolling circle amplification (dFC-LRCA)/miR into nono-flower balls (NFB)	Padlock DNA probe	10–4000 pM	miR21: 3.09 pM, MiR141: 1.58 pM, Let-7a: 1.34 pM	[72]
miR-155	Electrochemical sensor	AuNps modified screen-printed carbon electrode (SPCE) was an immobilized hairpin DNA probe	Complementary DNA	-	3.57 fM	[77]
miR-155, miR-21, and miR-16	Electrochemical	Reduced graphene oxide/poly(2-aminobenzylamine)/gold nanoparticles/porous hollow silver–gold nanoparticles (PHSGNPs) tagged with different metal ions	Complementary DNA	1 fM to 10 nM	MiR-155:0.98 fM, MiR21: 3.58 fM, MiR16: 0.25 fM	[79]
SAA1	Localized surface plasmon resonance (LSPR) and interferometry	Nanoporous anodic aluminum oxide (AAO) generated by two-step electrochemical anodization process. Immobilized SAA1 antibody on the gold coated AAO surface	Antibody	10 ag–1 µg/mL	100 ag/mL	[201]
SAA	Electrochemical biosensor	Multi-walled carbon nanotubes (MWCNTs), manganese oxide nanospheres (MnO_2_NSs), and cobalt oxide nanoparticles (Co_3_O_4_NPs) nanocomposite coated over the screen-printed electrode (SPE) by molecularly imprinted polymer technique	Molecularly imprinted polymer (MIP) for SAA	0.01 pM–1 µM	0.01 pM	[202]
SAA	Nephelometric immunoassay	Anti-SAA antibody-coated latex agglutination	Antibody	0.17–10 mg/mL	-	[203]
NSE	Electrochemical	Interaction of anti-NSE antibody with 5,10,15,20- tetra(4-aminophenyl) porphyrin (Zr-TAPP) complex.	Antibody	100 fg–2 ng/mL	7.1 fg/mL	[206]
NSE	Surface plasmon-enhanced fluorescence (SPF)	A mussel-inspired polydopamine (PDA) coated disposable silver plasmonic functionalized chip	Antibody	1 ng/mL to 100 ng/mL	0.5 ng/mL in buffer 1.4 ng/mL in human serum	[207]
NSE	Paper-based lateral flow strip (PLFS) and surface-enhanced Raman scattering (SERS)	Au nanostar, Raman Reporter, silica sandwich nanoparticles integrated nanocomposite	Antibody	1 ng/mL to 0.05 mg/mL	0.08 ng/mL	[208]
NSE	Electrochemical	Anti-Nse antibody immobilized on three-dimensional macroporous reduced graphene oxide/polyaniline (3DM rGO/PANI) film	Antibody	0.5 pg/mL to 10 ng/mL	0.1 pg/mL	[209]
NSE	Electrochemical	Separation of CuS-Ab2 filter from the large-size Fe3O4-Ab1/NSE/CuS-Ab2 immunocomplex; the free CuS-Ab2 undergo electron transfer processes between Cu^2+^ and Cu^+^ at the gold electrode surface and generate current	Antibody	100 fg/mL to 50 ng/mL	33 fg/mL	[210]
NSE	Surface plasmon resonance (SPR) based aptasensor	Anti-Nse aptamer immobilized on the SPR sensor chip	Aptamer	3.9 nM to 1 µM	3.9 nM	[105]
NSE	Chemiluminescent aptasensor	NSE complexed aptamer immobilized on the magnetic beads/primary mouse NSE antibody and alkaline phosphatase (ALP) labeled secondary goat antimouse antibodies/ALP triggered AMPPD chemiluminescence	Aptamer/antibody	1 to 100 ng/mL	0.1 ng/mL	[212]
NSE	Field-effect transistor (FET)	Anti-NSE antibody immobilized FET sensor chip	Antibody		10 ng/mL	[213]
SCCA	Electrochemiluminescence (ECL)	Magnetic graphene oxide (Fe3O4@GO)/AuNPs/g-C3N4	Antibody	0.001 to 10 ng/mL	0.4 pg/mL	[216]
SCCA	Electrochemiluminescence (ECL)	Molecular imprinting by lectropolymerization of ZnSe Qds encapsulated in Fe(III)-MIL-88B-NH2 and generated Fe(III)-MIL-88B H2@ZnSeQDs	Antibody	0.0001 to 100 ng/mL	31 fg/mL	[217]
SCCA	Reusable LSPR	Triangle-shaped silver nanoparticle array/nanosphere lithography/self-assembled monolayer (SAM) of 11-mercaptoundecanoic acid (MUA)	Antibody	0.1 to 1000 pM	0.128 pM	[218]
HSA70	Electrochemical immunosensor	Gold-coated plastic chip electrode (PCE) platform	Antibody	0.01–1000 ng/mL	3.5 pg/mL	[229]
HSP60	Quartz crystal microbalance (QCM)	QCM coated with HSP60 specific capture antibody and detection antibody of modified AuNps	Antibody	10 pg/mL–100 µg/mL	83 pg/mL	[230]
HSP70	Electrochemical	AuNp modified ITO coated polyethylene terephthalate (PET) electrodes	Antibody	1–166 fg/mL	0.0618 fg/mL	[231]

## Glossary

SCLC	Small-cell lung cancer
NSCLC	Non-small-cell lung cancer
RCTs	Randomized controlled clinical trails
TNMs	Tumors (Ts), nodes (Ns), and metastases (Ms)
TTF	Thyroid transcription factor
CT	Computed tomography
LDCT	Low-dose computed tomography
AI	Artificial intelligence
MRI	Magnetic resonance imaging
PET	Positron emission tomography
BRE	Bio-recognition element
EGFR	Epidermal growth factor receptor
NAATs	Nucleic acid amplification assays ()
ctDNA	Circulating tumor DNA
ssDNA	Single-strand DNA
dsDNA	Double-strand DNA
MiR	MicroRNA
cDNA	Complementary DNA
ERA	Exonuclease (Exo) III-assisted target recycling amplification
ECM	Extracellular matrix
PDACs	Pancreatic ductal adenocarcinomas
COPD	Chronic obstructive pulmonary disease
EBC	Exhaled breath condensate
ProGRP	Pro-gastrin-releasing peptide
CYFRA21-1	Cytokeratin 19 fragment 21-1
CEA	Carcinoembryonic antigen
IL-10	Interleukin-10
CA	Cancer antigen
VEGF	Vascular endothelial growth factor
ENO1	Enolase-1
β-HCG	Human chorionic gonadotropin
FBP	Folate-binding protein
FR	Folate receptor
AFP	Alpha-fetoprotein
SAA	Serum amyloid A
NSE	Neuron-specific enolase
M.Tase	Methyl transferase
GOD	Glucose oxidase
BSA	Bovine serum albumin
HA	Hyaluronic acid
HRP	Horseradish peroxidase
HDL	High-density lipoprotein
LDL	Low-density lipoprotein
CRP	C-reactive protein
CGA	Chromogranin A
ECL	Electrochemiluminescence
MEF	Metal-enhanced fluorescence
SPR	Surface plasmon resonance
LSPR	Localized surface plasmon resonance
LRET	Luminescence resonance energy transfer
FRET	Fluorescence resonance energy transfer
EC	Electrochemical
PEC	Photoelectrochemical
MESIA	Magnetic-force-assisted electrochemical sandwich immunoassay
ECL	Electrogenerated chemiluminescence
EIS	Electrochemical impedance spectroscopy
CL-ELISA	Chemiluminescence enzyme-linked immunosorbent assay
ELISA	Enzyme-linked immunosorbent assay
AlphaLISA	Amplified luminescent proximity homogeneous assay
FET	Field effect transistor
G-FET	Graphene field-effect transistor
poly-Si NW FET	Polycrystalline silicon nanowire field-effect transistor
µPAD	Microfluidic paper-based analytical device
LFIA	Lateral flow immunoassay
RCA	Rolling circle amplification
PCR	Polymerase chain reaction
qPCR	Quantitative polymerase chain reaction
RT-qPCR	Real-time quantitative polymerase chain reaction
NRCA	Netlike rolling circle amplification
LAMP	Loop-mediated isothermal amplification
BIND	Biomolecular interaction detection
SPE	Screen printing electrode
LSV	Linear-sweep voltammetry
ASV	Anodic stripping voltammetric
CV	Cyclic voltammetry
DPV	Differential pulse voltammetry
SPGE	Screen-printed graphene–carbon paste electrode
MOE	Molecular operating environment
PEC	Photoelectrochemical
MS-PCR	Methylation-specific polymerase chain reaction
MS-snuPE	Methylation-sensitive single nucleotide primer extension
MBD	Methyl-binding domain
HPLC	High-performance liquid chromatography
MS	Mass spectrometry
GC	Gas chromatography
GC-MS	Gas chromatography mass spectrometry
LC-MS	Liquid chromatography mass spectrometry
SERS	Surface-enhanced Raman scattering
SPF	Surface plasmon-enhanced fluorescence
PLFS	Paper-based lateral flow strip
LFA	Lateral flow assay
ASSURED	Affordable, specific, sensitive, user-friendly, rapid and robust, equipment-free, and deliverable to end user
MB	Methylene blue
Fc	Ferrocene
FA	Folic acid
ITO	Indium tin oxide
APTES	(3- aminopropyl)triethoxysilane
3-Th-COOH	3-thiophenecarboxylic acid
NMM	N-methylmesoporphyrin IX
TiO_2_ NPs	Titanium dioxide nanoparticles
AuNP	Gold nanoparticle
AuNC	Gold nanocube
AuNR	Gold nanorod
UCNPs	Upconversion nanoparticles
Ag NPs	Silver nanoparticles
CQDs	Carbon quantum dots
QDs	Quantum dots
GO	Graphene oxide
rGO	Reduced graphene oxide
GQD	Graphene quantum dots
GO-COOH	Carboxylated graphene oxide
CNTs	Carbon nanotubes
SPIONs	Super-paramagnetic iron oxide nanoparticles
IONPs	Iron oxide nanoparticles
PC	Photonic crystal
CuO NWs	Copper oxide nanowires
CuO Nps	Copper oxide nanoparticles
GMPs	Gold-capped magnetic nanoparticles
PtNPs	Platinum nanoparticles
SeNPs	Selenium nanoparticles
AAO	Anodic aluminum oxide
MWCNTs	Multiwalled carbon nanotubes
MNO_2_NSs	Manganese oxide nanospheres
PHSGNPs	Porous hollow silver–gold nanoparticles
Co_3_O_4_NPs	Cobalt oxide nanoparticles
PNA	Peptide nucleic acid
MCH	6-Mercaptohexanol
DTT	Dithothreitol
TMB	3,3′,5,5′-Tetramethylbenzidine
PhCN	Phthalocyanine
[Fe(CN)_6_]^3−^	Ferricyanide
[Fe(CN)_6_]^4−^	Ferrocyanide
Cys/GS-Nf	Cysteine/Nafion-graphene
PTCDA	3,4,9,10-Perylenetetracarboxylic dianhydride
MOF	Metal–organic frameworks
PETp	Polyethylene terephthalate
PPy	Polypyrrole
poly-TTCA	Poly(terthiophene carboxylic acid)
PDA	Polydopamine
PEG	Poly(ethylene glycol)
eATRP	Electrochemically induced atom transfer radical polymerization
CCP	Cationic conjugated polymers
MIP	Molecularly imprinted polymer
TESUD	11-(Triethoxysilyl) undecanal
PBASE	1-Pyrenebutyric acid N-hydroxysuccinimide ester
GA	Glutaraldehyde
Zr-TAPP	5,10,15,20-Tetra(4-aminophenyl) porphyrin
EDC	1-Ethyl-3-(3-dimethylaminopropyl)carbodiimide
NHS	N-hydroxysuccinimide
LOD	Limit of detection
POCT	Point-of-care testing
FAM	6-Carboxyfluorescein
HSPs	Heat shock proteins
